# Temperature dependent CO_2_ behavior in microporous 1-D channels of a metal-organic framework with multiple interaction sites

**DOI:** 10.1038/srep41447

**Published:** 2017-01-27

**Authors:** Dongwook Kim, Jaehun Park, Yung Sam Kim, Myoung Soo Lah

**Affiliations:** 1 Department of Chemistry, UNIST, Ulsan 44919, Korea; 2 Pohang Accelerator Laboratory, POSTECH, Pohang 37673, Korea

## Abstract

The MOF with the encapsulated CO_2_ molecule shows that the CO_2_ molecule is ligated to the unsaturated Cu(II) sites in the cage using its Lewis basic oxygen atom via an angular η^1^-(O_A_) coordination mode and also interacts with Lewis basic nitrogen atoms of the tetrazole ligands using its Lewis acidic carbon atom. Temperature dependent structure analyses indicate the simultaneous weakening of both interactions as temperature increases. Infrared spectroscopy of the MOF confirmed that the CO_2_ interaction with the framework is temperature dependent. The strength of the interaction is correlated to the separation of the two bending peaks of the bound CO_2_ rather than the frequency shift of the asymmetric stretching peak from that of free CO_2_. The encapsulated CO_2_ in the cage is weakly interacting with the framework at around ambient temperatures and can have proper orientation for wiggling out of the cage through the narrow portals so that the reversible uptake can take place. On the other hand, the CO_2_ in the cage is restrained at a specific orientation at 195 K since it interacts with the framework strong enough using the multiple interaction sites so that adsorption process is slightly restricted and desorption process is almost clogged.

Recent global warming is closely related to the increased CO_2_ concentration in the atmosphere^1^. One way to tackle the problem is CO_2_ capture-storage and utilization^2^,^3^,^4^,^5^. Microporous metal-organic frameworks (MOFs) received great attentions for CO_2_ capture and utilization^6^,^7^,^8^,^9^,^10^,^11^,^12^. The properties of a MOF can be modulated to meet necessities for CO_2_ sequestration agents using not only its organic residues but also metal ions in pore surface.

Though there are many structural reports on the CO_2_ bound species, most of them are of molecular complexes that contain metal sites interacting with CO_2_ in strong coordination bond such as η^2^-(C, O)^5^, η^1^-(C)^6^ and linear η^1^-(O_L_) coordination modes^13^ (Fig. 1). There are also several reports on the structural characteristics of the CO_2_ encapsulated in micropores of MOFs^13^,^14^,^15^,^16^,^17^,^18^,^19^,^20^,^21^, however, the interactions are weak non-covalent interactions, such as hydrogen bonding, dipole-quadrupole interaction and van der Waals interactions (C-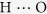 and O-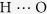 interactions), only with organic residues in the pore surface.

The structural information of the encapsulated CO_2_ interacting with the framework using multiple binding sites is scarce^22^. On the other hand, while there are several reports on the vibrational mode analysis of CO_2_ strongly bound to metal centers^13^,^23^,^24^,^25^,^26^,^27^, only a few investigations on the correlation between the vibrational modes of the encapsulated CO_2_ weakly interacting with the framework and its structural data are reported^16^,^17^,^18^,^19^,^22^,^28^,^29^.

Here, we report the temperature dependent single crystal structure analyses of a microporous MOF with bound CO_2_ in microporous 1-D channels via both a weak angular η^1^-(O_A_) coordination of CO_2_ to Cu(II) sites and a dipole-quadrupole interaction between a Lewis basic atom of a framework and the Lewis acidic carbon atom of CO_2_. Temperature dependent vibrational modes of bound CO_2_ are investigated using infrared (IR) spectroscopy together with temperature dependent crystal structures with the encapsulated CO_2_. Reversible CO_2_ uptakes at around ambient temperatures but irreversible CO_2_ uptake at 195 K could be explained based on temperature dependent structural information and vibrational band analyses of the bound CO_2_.

## Results and Discussion

### Single crystal structure analyses of the MOF and its CO_2_-bound MOF derivatives

**Figure 2:**
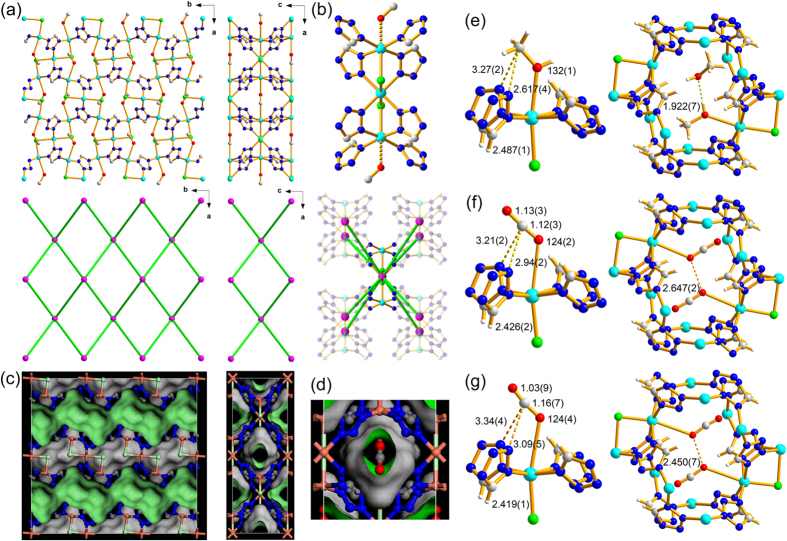
(**a**) Ball-and-stick diagrams of **1** viewed along the crystallographic *c*-axis (left) and *b*-axis (right), respectively, where [Cu_3_Cl_2_] SBU as 8-c nodes are represented by pink spheres and the linkers between the 8-c nodes are represented by the green sticks. (**b**) A [Cu_3_Cl_2_(tz)_8_] cluster (top) and the [Cu_3_Cl_2_] SBU as an 8-c node (bottom). (**c**) The space-filling diagrams of **1** drawn as the same view as the ball-and-stick diagrams in (**a**). (**d**) A 1-D channel viewed along the crystallographic *b*-axis, where a CO_2_ is ligated to Cu(II) site. Ball-and-stick diagrams of the single crystal structures of (**e**) MeOH-bound **1a** at 173 K, (**f**) CO_2_-bound **1a** at 195 K, and (**g**) CO_2_-bound **1a** at 296 K.

A solvothermal reaction of CuCl_2_ with tetrazole (tz) in methanol (MeOH)/acetonitrile mixed solvent produced block-shaped blue crystalline product of the formula unit, Cu_3_Cl_2_(tz)_4_(MeOH)_2_ ((**MeOH)**_**2**_**@1a**). A single crystal structure analysis revealed that the product is a 3-D MOF of an 8-c **bcu** topology^30^ with linear trinuclear [Cu_3_Cl_2_] secondary building unit (SBU) as an 8-c node and tridentate tz as a ditopic linker (Fig. 2). A partially occupied methanol molecule is weakly ligated at the Jahn–Teller elongated sixth coordination site of the terminal copper atom (Cu1–O1M: 2.617(4) Å) of the linear trinuclear [Cu_3_Cl_2_] SBU (Fig. 2e).

**1** has potential 1-D microporous channels made of cages aligned along the crystallographic *b*-axis (Fig. 2a and c). The shortest and the longest dimensions of the cage are ~3.5 and 5.5 Å, respectively, and the neck-like portal window of ~2.5 Å in diameter is formed between the cages (Figs 2c and S1). Each cage contains two methanol molecules. An approximate half of them in ~0.5 site occupancy are ligated to the two symmetry-related Cu(II) sites in the cage (Figs 2e and S2a) and the remaining lattice methanol molecules are statistically disordered in the vicinity of the ligated methanol molecules (Figs 2e and S2b).

The properly activated sample, **1a**, was prepared by vacuuming (~10^−2^ torr) (**MeOH)**_**2**_**@1a** at 160 °C for 7 d (Figs S3–S7). Single crystals of the CO_2_-bound **1a** ((**CO**_**2**_)_**0.8**_**@1a-195K**) were obtained by keeping single crystals of **1a** in CO_2_ at ~1.5 bar and at ambient temperature for an hour, and then cooling down to 195 K. The structure analysis revealed that CO_2_ was ligated to unsaturated metal site using its Lewis basic oxygen atom in an angular η^1^-(O_A_) coordination mode with a 0.40(1) site occupancy factor (0.8 CO_2_ molecule per cage) (Figs 2f and S8). The Cu−O_CO2_ distance, 2.94(2) Å, is slightly longer than the Cu−O_MeOH_ distance, 2.617(4) Å, of the bound MeOH in the as-synthesized crystal but still significantly shorter than the sum of their van der Waals radii sum (3.55 Å)^14^. The Cu−O_CO2_ distance is, much longer than the corresponding Ni-O_CO2_ bond distances in Ni-MOF-74 structure obtained from the PXRD experiment, 2.29(2) Å^22^, the corresponding Mg−O_CO2_ bond distances in Mg-MOF-74 structures obtained from the NPD experiments, from 2.24(3) Å to 2.39(6) Å^31^, but is comparable to that of Cu-MOF-74, 2.86(3) Å^32^. It is well-known that the Lewis acidic carbon atom of the CO_2_ molecule can interact with the Lewis basic nitrogen atom in the pore environment^16^,^17^,^18^,^19^,^29^. Similar Lewis acid-base interaction is also observed in the CO_2_-bound **1a**. The Lewis acidic carbon atom (C1C) of the bound CO_2_ molecule is interacting with the two Lewis basic nitrogen atoms (N3) of two symmetry-related tz ligands. The distance between C1C and N3 (3.21(2) Å) is slightly shorter than their van der Waals radii sum (3.30 Å). The Cu2-O1C-C1C bond angle (124(2)°) is in the range those in Ni-MOF-74, Mg-MOF-74, and Cu-MOF-74 structures (from 117(2) to 144(2)°). The observed site occupancy factor of the ligated CO_2_ molecule, 0.40(1), is due to steric repulsion between the CO_2_ molecules bound to the symmetry-related Cu(II) sites in a cage. The interatomic distance between the symmetry-related ligated oxygen atoms of the bound CO_2_ molecules (2.647(2) Å) is much closer than their van der Waals radii sum (3.1 Å) (Fig. 2f). The steric repulsion between the CO_2_ molecules hinders simultaneous ligation of two CO_2_ molecules in the same cage. The geometry of the bound CO_2_ at 195 K is similar to that of a reported free CO_2_ structure. The C−O bond distances, 1.12(3) Å and 1.13(3) Å, in the bound CO_2_ structure are not distinguishable to that in the free CO_2_ structure, 1.155(1) Å^33^. The little variation in the C−O bond distance in the bound CO_2_ is due to the combined effect of two different interactions between the CO_2_ molecule and the framework. The CO_2_ molecule donates its σ electron to the metal center through the ligating oxygen atom but simultaneously accepts σ electron from the two Lewis basic nitrogen atoms of the tz ligands through the Lewis acidic carbon atom of the bound CO_2_. The O−C−O bond angle in the bound CO_2_ structure, 176(3)°, is also nearly linear as that in the free CO_2_ structure.

The single crystal of (**CO**_**2**_)_**0.26**_**(H**_**2**_**O)**_**0.15**_**@1a-296K-5h** (the single crystal of **CO**_**2**_**@1a-195K** stood at ambient condition for five hours) showed the bound CO_2_ molecule in the cage with reduced site occupancy, 0.13(2) (Fig. 2g). Though the CO_2_ molecule is still ligated to the metal center, the Cu−O bond distance of (**CO**_**2**_)_**0.26**_**(H**_**2**_**O)**_**0.15**_**@1a-296K-5h** at 296 K, 3.09(5) Å, is elongated by ~0.15 Å than that of (**CO**_**2**_)_**0.8**_**@1a-195K** at 195 K but is still slightly shorter than the van der Waals sum of Cu and O atoms. However, interestingly, the Lewis acidic carbon atom (C1C) of the bound CO_2_ molecule is no longer interacting with the Lewis basic nitrogen atom (N3) of the ligand. The distance between C1C and N3 (3.34(4) Å) is slightly longer than their van der Waals radii sum, 3.30 Å. The complete replacement of the bound CO_2_ molecules in the pore by the water molecules in air took 10 days at ambient condition (Figs S8 and S9).

### The vibrational mode analysis of the CO_2_ molecule encapsulated in a cage

**Figure 3:**
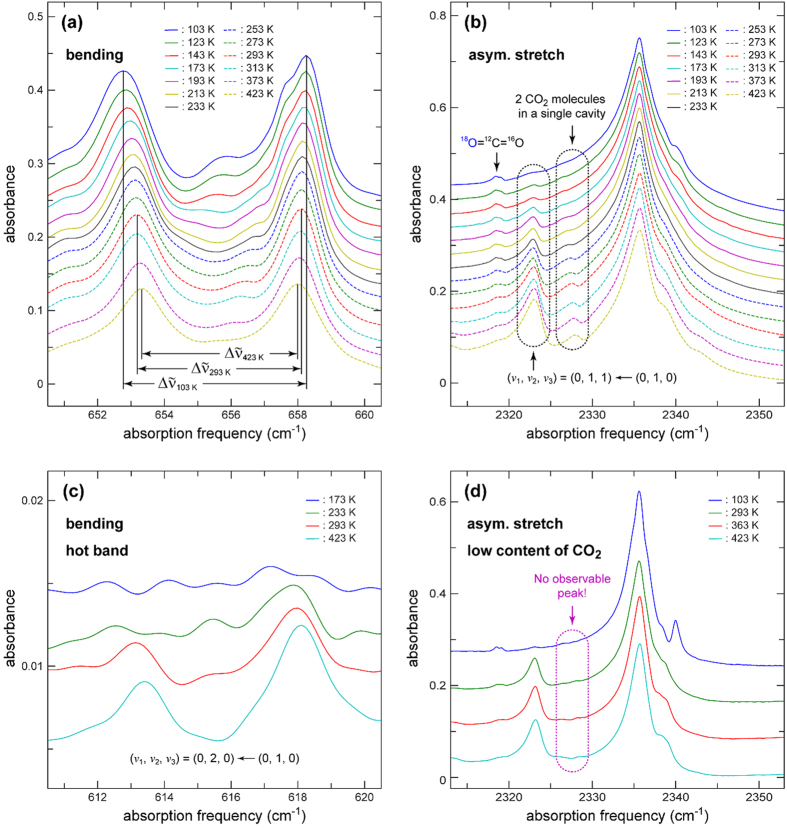
Temperature- and CO_2_ concentration-dependent FTIR spectra of 1a filled with ^12^CO_2_. (**a**) Spectra in the bending region of ^12^CO_2_. Δ
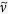
_103 K_ (5.49 cm^−1^), Δ
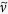
_293 K_ (4.92 cm^−1^), and Δ
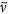
_423 K_ (4.67 cm^−1^) represent the separation between the two peak maxima at 103, 293, and 423 K, respectively. Similar spectra are reported for **1a** filled with ^13^CO_2_ in Fig. S11. (**b**) Spectra in the asymmetric stretch region of ^12^CO_2_. The peaks enclosed with rounded rectangles in the spectral region of ~2323 and ~2327 cm^−1^ represent a vibrational hot band (starting from the first excited state of the bending mode) and a peak originating from the existence of 2 CO_2_ molecules in a single cavity, respectively. (**c**) Spectra in the spectral region of *v*_2_ = 1 → *v*_2_ = 2 transition (hot band). (**d**) Spectra of **1a** with low CO_2_ content in the spectral region of the asymmetric stretch. Note that the spectra in (**a**,**b** and **c**) were obtained from the same sample (high content of CO_2_), whereas the spectra in (**d**) were obtained from a different sample (low content of CO_2_).

Figure 3 shows temperature-dependent infra-red (IR) spectra of the MOF, **1a**, filled with ^12^CO_2_ molecules in the bending and asymmetric stretch regions (see Fig. S10 for whole-range spectra). In the IR spectra of the bending region given in Fig. 3a, two separate peaks are observed, which is distinctively different from the case of a free CO_2_ molecule where only a single bending peak appears because its two bending modes are degenerate. This observation suggests that the interaction between a CO_2_ molecule and **1a** disturbs its bending motion to generate two separate peaks. The center frequency of the two peaks is also red-shifted by 11.7 cm^−1^ compared to the degenerate bending frequency of a free CO_2_ of 667.3 cm^−1^ 34. Similar splitting of the bending modes and red-shift of the center frequency has been reported in the IR spectra of the CO_2_ bound in an η^1^-(O_A_) coordination mode in Ni-MOF-74^10^. The splitting of the two bending modes must be related to the binding energy of a CO_2_ molecule to the framework and a larger separation indicates stronger interaction between them. The separation between the two peaks decreases as the temperature increases from 5.49 cm^−1^ at 103 K to 4.92 and 4.67 cm^−1^, respectively, at 293 and 423 K. Provided that the binding energy is proportional to the separation, the binding energy at 293 and 423 K, respectively, decreases by 11 and 15% compared with that at 103 K. The CO_2_ molecule encapsulated in the cage is less strongly bound to the framework at elevated temperatures. These observations are in agreement with the temperature dependent crystal structures of CO_2_@**1a**. Though the bound CO_2_ structures themselves from the temperature dependent single crystal structure study are not as sensitive as the bending modes of the bound CO_2_ molecule, the temperature dependent interactions between the CO_2_ and the framework are in good agreement with the temperature dependent bending modes of the bound CO_2_ molecule. While the peak separation of the two symmetric bending modes of the bound CO_2_ molecule is temperature dependent, the center frequency is insensitive to temperature. Temperature insensitivity of the center frequency is due to the combined result of simultaneous weakening of both the sigma electron donation to the Cu(II) ion through the Lewis basic oxygen atom of the bound CO_2_ and the sigma electron acceptance from the nitrogen atoms of the tz ligands via the Lewis acidic carbon atom of the bound CO_2_ molecule.

Figure 3b shows that the main asymmetric stretching band at ~2335 cm^−1^ with a FWHM of ~7 cm^−1^ is red-shifted by 14 cm^−1^ compared to the corresponding band of free ^12^CO_2_ at 2349 cm^−1^ 33. The red-shift indicates that the σ electron donation through the Lewis basic oxygen atom of the bound CO_2_ to the Cu(II) center is slightly stronger than the σ electron acceptance through the carbon atom of the bound CO_2_ from the nitrogen atoms of the tz ligands. An asymmetric stretching band shift is not a good indicator of the binding strength of an encapsulated CO_2_ to a framework. It is a combined effect of electron donation and acceptance of the encapsulated CO_2_ to and from the framework. The encapsulated CO_2_ in the series of isostructural MOFs, M-MOF-74 (where, M = Ni(II), Mg(II), Zn(II), and Co(II)), showed different shifts of the asymmetric CO_2_ stretching band depending on metal ions even though the frameworks are isostructural^35^.

The side bands at the high-frequency side are likely to originate from slightly different local structures of CO_2_ inside the cage. At the lower-frequency side of the main band, two peaks, each of which is enclosed with a rounded rectangle, are observed. The temperature dependent peak at ~2323 cm^−1^ was assigned as a vibrational hot band coming from the transition, (ν_1_, ν_2_, ν_3_) = (0, 1, 0) → (0, 2, 0)^36^. The assignment is further clarified by the hot-band spectra in the bending region shown in Fig. 3c, where the magnitude of the peaks follows the Boltzmann distribution. However, the peak at ~2327 cm^−1^, which shows similar temperature dependence as the peak at ~2323 cm^−1^, was assigned to be a peak arising from CO_2_ double occupancy in a single cage (two CO_2_ molecules in a single cage interacting to each other). Figure 3d shows the asymmetric stretch region of another sample that contains less amount of CO_2_ by a factor of 3. The integrated areas of the peaks in the spectra of the low CO_2_ content sample are about 1/3 of those of their corresponding spectra (Fig. S12). The double-occupancy peak is hardly observable at 2327 cm^−1^ indicating that the integrated area of the peak is not linearly proportional to CO_2_ concentration and the peak must be from the double occupancy. In the spectra of the low CO_2_ content sample, the peaks are narrower than their corresponding ones in the high-content spectra. This observation suggests that the skeleton of **1a** is rather flexible and the structural distribution of the encapsulated CO_2_ becomes more inhomogeneous with increase in CO_2_ content^37^.

The observation that the magnitude of the double-occupancy peak increases as the temperature increases (Fig. 3b) should also be paid attention to. A single CO_2_ molecule in a single cage is preferred at lower temperature under lower concentration of CO_2_. The steric repulsion between the CO_2_ molecules ligated to the two symmetry-related metal sites in the same cage at low temperature is responsible for this single occupancy. Two CO_2_ molecules in a single cage are allowed at high temperature under higher CO_2_ content. The reduced restraint on the position of the weakly interacting CO_2_ molecule in the cage at high temperature allows two CO_2_ molecules in a single cage. The existence of the double-occupancy peak helps us to understand the process of CO_2_ filling into the 1-D microporous channels, made of small cages interlinked with small neck-like portal windows. Not only a *hopping process* of a CO_2_ molecule into an empty cage but also a *filling process* of a second CO_2_ molecule into the cage with an encapsulated CO_2_ are operating.

### CO_2_ sorption behaviors

**Figure 4:**
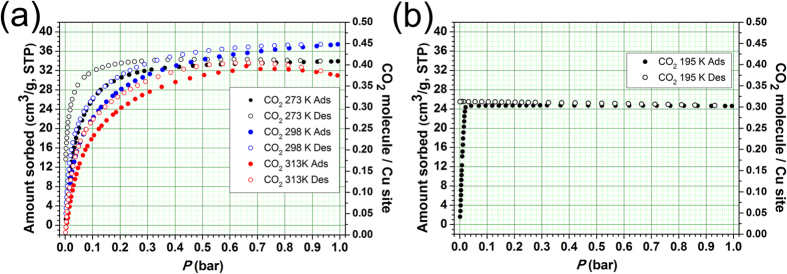
Gas sorption of 1a. (**a**) Reversible CO_2_ adsorption and desorption isotherms at 273 K, 298 K, and 313 K. (**b**) Irreversible CO_2_ adsorption and desorption isotherms at 195 K.

Even though **1a** had microporous 1-D channel, it does not show any N_2_ (at 77 K and 308 K), H_2_ (at 77 K) and CH_4_ (at 195 K and 308 K) adsorptions (Fig. S13). However, **1a** shows reversible CO_2_ uptake at 273 K even though the kinetic diameter of CO_2_ (3.3 Å) is much larger than the dimension of the portal window and the isotherms are of a typical type I (Fig. 4a). The large quadruple moment of CO_2_ might be responsible for such uptake behavior. The CO_2_ molecule can interact with the framework strong enough to induce the portal dimension of the 1-D microporous channels large enough for the reversible CO_2_ adsorption and desorption. The total CO_2_ uptake amount of **1a** at 273 K and 1 bar, 33.9 cm^3^/g, corresponds to 0.82 CO_2_ molecules per cage (or 0.41 CO_2_ molecules per unsaturated Cu(II) center). The CO_2_ sorption isotherms of **1a** at 298 K are also reversible type I isotherms and the total CO_2_ uptake amount at ~1 bar is 37.4 cm^3^/g, which is slightly larger than the CO_2_ uptake amount at 273 K and ~1 bar. The increased CO_2_ uptake amount at the higher temperature is due to increased double occupancy of CO_2_ molecules in a single cage and subsequent pore filling up to the cages located at the inner parts of the microporous 1-D channels. The total CO_2_ uptake amount at 313 K and ~1 bar, 31.3 cm^3^/g, is slightly smaller than that at 298 K. The reduced CO_2_ uptake amount is due to decreased CO_2_-framework interaction at the higher temperature. The adsorption enthalpy (−ΔH_ads_) of CO_2_ on **1a** calculated using the adsorption isotherms at 298 K and 313 K is −32– −25 kJ/mol (Fig. S14), which will be the lower bound because the isotherms obtained are not in the true equilibrium condition due to slow adsorption kinetics of CO_2_. The adsorption enthalpy (−ΔH_ads_) of CO_2_ estimated using desorption isotherms at 298 K and 313 K is −41– −35 kJ/mol, which is comparable to the CO_2_ adsorption enthalpy on Mg-MOF-74, ~40 kJ/mol^22^,^32^, but is much larger than that on Cu-MOF-74, ~22.1 kJ/mol^32^.

Interestingly, the CO_2_ sorption on **1a** at 195 K is irreversible (Fig. 4b). The CO_2_ adsorption isotherm at 195 K shows steep rise at very low pressure. The adsorption reaches to its maximum amount, 24.3 cm^3^/g, at 0.022 bar. The amount of adsorbed CO_2_ corresponds to ~0.60 CO_2_ molecule per cage (or ~0.30 CO_2_ molecule per unsaturated metal site), which is much smaller than the expected maximum amount, one CO_2_ molecule per cage (or 0.5 CO_2_ molecule per unsaturated metal site). Significant portions of the inner cages in the 1-D porous channel may not be accessible for CO_2_ molecule because the channels are already blocked by the CO_2_ molecules bound strongly to the framework as shown in Fig. 2d. There is no indication of CO_2_ desorption even at 0.0015 bar.

At high temperature, not only *hopping process* of a CO_2_ molecule into an empty cage but also *filling process* of a second CO_2_ molecule into the cage with an encapsulated CO_2_ molecule are allowed so that even most of the empty inner cages of the 1-D channels could be occupied with CO_2_ molecules and the CO_2_ molecules in the inner cages the 1-D channels can also be emptied via a reverse process. However, at low temperature, the filling of a second CO_2_ molecule into the cage with an encapsulated CO_2_ molecule is severely hindered because the CO_2_ molecule bound strongly at the open metal center does not allow the second CO_2_ molecule into the same cage. At low temperature, the main pore filling is via *hopping process* so that the maximum amount of CO_2_ captured in the 1-D channels is significantly smaller than the expected maximum amount of CO_2_. When multiple consecutive cages in a 1-D channel are filled with CO_2_ molecules, desorption of CO_2_ is also severely hindered, which is the cause of the irreversible CO_2_ sorption behavior of **1a** at 195 K.

## Conclusions

Although each cage contains two unsaturated Cu(II) sites and the cage size is large enough for two CO_2_ molecules, the CO_2_ uptake amount is only ~0.9 CO_2_ molecule per cage at 298 K and 1 bar, which is due to the steric repulsion between the two bound CO_2_ molecules in the cage. Temperature dependent single crystal structure analyses revealed that the interaction between the bound CO_2_ molecule and the framework of the MOF is temperature-dependent. As temperature increases, both the CO_2_ coordination to the unsaturated Cu(II) center and the Lewis acid−base interaction between the carbon atom of the CO_2_ molecule and the nitrogen atom of the ligand are simultaneously weakened.

The red-shifted vibrational peaks of the bound CO_2_ molecule indicate that the electron donation to the metal center through the oxygen atom of the CO_2_ molecule is slightly larger than the electron acceptance from the nitrogen atom of the ligand through the carbon atom of the CO_2_ molecule. The temperature dependent separation of the two bending peaks also supports the temperature dependent interaction between the CO_2_ molecule and the framework of the MOF. As temperature increases, the separation of the bending peaks decreases. The observation tells that the CO_2_–framework interaction decreases as the temperature increases as observed in the temperature dependent structure analyses of a single crystal of CO_2_@**1a**.

The reversible CO_2_ uptakes on **1a** were observed at 298 and 313 K, respectively, even though the portal dimension of the cage aligned along the microporous 1-D channel is smaller than the kinetic diameter of CO_2_ molecule. The CO_2_ uptake amount per unsaturated Cu(II) center at 298 K, 1 bar is 0.45 molecule, which is only slightly smaller than the probable maximum value, 0.5. On the other hand, the irreversible CO_2_ uptake was observed at 195 K and the CO_2_ uptake amount per unsaturated Cu(II) center is only 0.30 molecule, which is much smaller than the expected value, 0.5. The decreased uptake amount at the lower temperature is due to the strongly interacting CO_2_ molecules in the cages aligned along the microporous 1-D channel hindering the other CO_2_ molecules to access the available inner cages in the 1-D channel. The different uptake behaviour at different temperature is related to both the small portal dimension of the cages aligned along the 1-D channel and the different strength of CO_2_−framework interaction in the cage. The reversible CO_2_ uptakes were observed at the higher temperature since the CO_2_ molecule interacting weakly with the framework could have proper orientation for the escape from the cavity through the small portal of the cages. However, the irreversible CO_2_ uptake was observed at the lower temperature since the CO_2_ molecule interacting strongly enough with the metal center in the cage could not have proper orientation for the escape from the cavity through the small portal.

## Methods

### Synthesis of the MOF, Cu_3_Cl_2_(tz)_4_(MeOH)_2_, 1

A 27.3 mg amount of CuCl_2_ (0.203 mmol) was dissolved in 4 mL MeOH in a 10 mL vial, and then 0.90 mL 0.45 M tetrazole (tz) acetonitrile solution (0.41 mmol) was added into the solution. The Teflon-sealed vial was heated to 70 °C for 7 days and then slowly cooled down to ambient temperature. The block-shaped blue crystals obtained were washed using 10 mL fresh methanol and filtered.

### IR spectroscopy

The samples were in the form of a KBr pellet of a ~200-μm thickness and a 13-mm diameter. The samples were located in a cell composed of two circular KBr windows of a 25-mm diameter. The windows were separated by a 200-μm thick spacer with 25 and 20 mm outer and inner diameters, respectively. FTIR spectra were recorded by a Varian 7000e FTIR spectrometer. The samples with KBr windows were placed in a home-made oxygen-free high thermal conductivity (OFHC) copper cell, and the temperature of the samples was controlled and maintained in a variable temperature cell holder (GS21525, Specac, UK). Details of the temperature-controllable cell assembly have been reported elsewhere^38^.

### Gas sorption measurements

All gas sorption isotherms were measured using a BELSORP-max (BEL Japan, Inc.) with a standard volumetric technique.

## Additional Information

**How to cite this article:** Kim, D. *et al*. Temperature dependent CO_2_ behavior in microporous 1-D channels of a metal-organic framework with multiple interaction sites. *Sci. Rep.* **7**, 41447; doi: 10.1038/srep41447 (2017).

**Publisher's note:** Springer Nature remains neutral with regard to jurisdictional claims in published maps and institutional affiliations.

## Supplementary Material

Supplementary Information

## Figures and Tables

**Figure 1:**
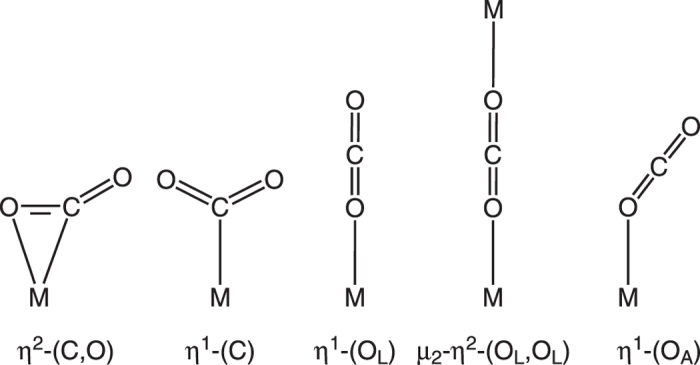
Coordination modes of CO_2_ to metal center.
