# Supplementary material for: Temperature dependent CO2 behavior in microporous 1-D channels of a metal-organic framework with multiple interaction sites
Source: Sci Rep. 2017 Jan 27;7:41447. doi: 10.1038/srep41447 (PMC5269755; doi:10.1038/srep41447)
Supplement: Supplementary Information [file srep41447-s1.doc]

<Supplementary information>

Temperature dependent CO_2_ behavior in microporous 1-D channels of a metal-organic framework with multiple interaction sites

Dongwook Kim,^†^ Jaehun Park,^‡^ Yung Sam Kim^*†^ and Myoung Soo Lah^*†^

^†^Department of Chemistry, UNIST, Ulsan 44919, Korea

^‡^Pohang Accelerator Laboratory, POSTECH, Pohang 37673, Korea

E-mail: mslah@unist.ac.kr; kimys@unist.ac.kr

Experimental section

**General procedures.** All reagents were purchased from commercial sources and were used without further purification. Elemental analysis (EA) for C, H, and N was performed using a Thermo Scientific Flash 2000 elemental analyzer. Fourier transform–infrared (FT–IR) spectra were recorded as KBr pellets with a Varian 7000e FT–IR spectrophotometer (4000–400 cm^–1^). Powder X-ray diffraction (PXRD) data were recorded using a Bruker D2 Phaser automated diffractometer at room temperature with a step size of 0.02° in 2θ angle. Simulated PXRD patterns were calculated with the Materials Studio program using single-crystal diffraction data.^S1^ Thermogravimetric analysis (TGA) was performed using a SDT Q600 instrument (TA Instruments, USA) under N_2_ with a heating rate of 5 °C min^–1^ between an ambient temperature and 600 °C.

**Synthesis of the MOF, Cu_3_Cl_2_(tz)_4_(MeOH)_2_, 1**. A 27.3 mg amount of CuCl_2_ (0.203 mmol) was dissolved in 4 mL methanol (MeOH) in a 10 mL vial, and then 0.90 mL 0.45 M tetrazole (tz) acetonitrile solution (0.41 mmol) was added into the solution. The Teflon-sealed vial was heated to 70 °C for 7 days and then slowly cooled down to ambient temperature. The block-shaped blue crystals obtained were washed using 10 mL fresh methanol and filtered. Yield: 26.3 mg, 66.0%. EA for **1**, Cu_3_Cl_2_(tz)_4_(MeOH)_2_ (C_6_H_12_N_16_O_2_Cl_2_Cu_3_, fw = 601.81 g/mol). Calculated: C, 11.97; H, 2.01; N, 37.24%; Found: C, 11.87; H, 2.00; N, 37.61%. IR (KBr, cm^–1^): 3481 (s, sh), 3481 (vs, br), 3094 (s), 2988 (w), 2954 (w), 2922 (m), 2821(m), 2712 (vw), 2566 (vw), 2434 (vw), 2363 (w), 2342 (w), 2293 (w), 2185 (vw), 2041 (vw), 1834 (w), 1818 (vw, sh), 1636 (m), 1463 (w, sh), 1447 (s), 1411 (m), 1380 (w), 1335 (w, sh), 1273 (s), 1170 (vw, sh), 1136 (s), 1122 (s), 1053 (m, sh), 1029 (s), 1015 (s), 922 (m), 694 (s), 606 (w, br), 436 (vw), 418 (w). The activated sample, **1a**, was prepared by vacuuming (~ 10^−2^ torr) the as-synthesized sample at 160 °C for 7 d. EA for **1a**, [Cu_3_Cl_2_(tz)_4_] (C_4_H_4_N_16_Cl_2_Cu_3_, fw = 537.73 g/mol). Calculated: C, 8.93; H, 0.75; N, 41.68%; Found: C, 9.00; H, 0.86; N, 41.64%. IR (KBr, cm^–1^): 3650 (w), 3553 (w), 3493 (w), 3416 (w), 3212 (w), 3094 (vs), 2886 (m), 2714 (w), 2589 (w), 2434 (w), 2399 (w), 2335 (m), 2292 (w), 2192 (vw), 1839 (m), 1614 (m), 1453 (s), 1338 (m), 1174 (w, sh), 1142 (s), 1122 (s), 1055 (m), 1032 (s), 923 (s), 769 (w), 694 (s), 654 (w), 507 (vw), 454 (vw), 415 (vw).

**Crystallographic data collection and refinement of the structure.** The diffraction data of a single crystal of **1** coated with Paratone oil were measured at 173 K with Mo Kα radiation on an X-ray diffraction camera system using an imaging plate equipped with a graphite crystal incident beam monochromator. A single crystal of CO_2_-bound **1a** was mounted on a capillary glass tip using epoxy glue and the diffraction data of the crystal were measured at 195 K with Mo Kα radiation on an X-ray diffraction camera system using an imaging plate equipped with a graphite crystal incident beam monochromator. The diffraction data were measured at 296 K using the same crystal five hours, one day, five days and ten days later on, respectively. The Rapid Auto software^S2^ was used for data collection and data processing. The diffraction data of a single crystal of **1a** mounted in a capillary under vacuum were measured at 393 K with synchrotron radiation on an ADSC Quantum-210 detector at 2D SMC with a silicon (111) double crystal monochromator (DCM) at the Pohang Accelerator Laboratory, Korea. The ADSC Q210 ADX program^S3^ was used for data collection, and HKL3000^S4^ was used for cell refinement, reduction, and absorption correction. The crystal structures were solved by the direct method with the SHELXTL-XS program and refined by full-matrix least-squares calculations with the SHELXTL-XL program package (Ver. 2008).^S5^

**1 ((MeOH)_2_@1a).** [Cu_3_Cl_2_(tz)_4_(MeOH)]∙MeOH, (C_6_H_12_N_16_O_2_Cl_2_Cu_3_), fw = 601.84 g∙mol^–1^, orthorhombic, space group *Pnnm*, *a* = 11.584(2) Å, *b* = 8.593(2) Å, *c* = 8.866(2) Å, *V* = 882.5(3) Å^3^, *Z* = 2, *μ* (Mo Kα, *λ* = 0.71073 Å) = 3.932 mm^–1^, T = 173(2) K, 8,176 reflections were collected, 1,075 were unique (*R*_int_ = 0.0275). One copper atom on a crystallographic 2/m symmetry site (Wyckoff site symmetry *a*), the other copper atom and one bridging chloride on a crystallographic mirror plane (Wyckoff site symmetry *g*) and a tz ligand on general position are observed as an asymmetric unit. Two methanol sites around a copper ion on the crystallographic mirror plane were identified in the difference Fourier map and were treated as statistically disordered methanol sites. The site occupancy factors were refined to 0.251(3) and 0.249(3), respectively. All non-hydrogen atoms are refined anisotropically; the hydrogen atom attached to the carbon atom of the tz ligand was found in the difference Fourier map and refined isotropically. The hydrogen atoms of the statistically disordered methanol were assigned isotropic displacement coefficient U(H) = 1.5U (C_Methyl_ and O_Hydroxyl_) and their coordinate were allowed to ride on the corresponding atom. Least-squares refinement of the structure converged at a final *R*1 = 0.0192, *wR*2 = 0.0449 for 1026 reflections with *I* > 2*σ*(*I*); *R*1 = 0.0204, *wR*2 = 0.0455 for all 1075 reflections. The largest difference peak and hole were 0.416 and −0.524 e·Å^−3^, respectively.

**1a.** [Cu_3_Cl_2_(tz)_4_] (C_4_H_4_N_16_Cl_2_Cu_3_), fw = 537.75 g∙mol^–1^, orthorhombic, space group *Pnnm*, *a* = 11.729(2) Å, *b* = 8.591(2) Å, *c* = 8.765(2) Å, *V* = 883.2(3) Å^3^, *Z* = 2, *μ* (synchrotron, *λ* = 0.62999 Å) = 2.814 mm^–1^, T = 393(2) K, 11,893 reflections were collected, 1,774 were unique (*R*_int_ = 0.0454). One copper atom on a crystallographic 2/m symmetry site, the other copper atom and one bridging chloride on a crystallographic mirror plane and a tz ligand on general position are observed as an asymmetric unit. All non-hydrogen atoms are refined anisotropically; the hydrogen atom attached to the carbon atom of the tz ligand was found in the difference Fourier map and refined isotropically. Least-squares refinement of the structure converged at a final *R*1 = 0.0411, *wR*2 = 0.1117 for 1724 reflections with *I* > 2*σ*(*I*); *R*1 = 0.0445, *wR*2 = 0.1214 for all 1774 reflections. The largest difference peak and hole were 1.640 and −1.687 e·Å^−3^, respectively.

(**CO_2_)_0.8_**@**1a-195K.** [Cu_3_Cl_2_(tz)_4_(CO_2_)_0.80_] (C_4.80_H_4_N_16_O_1.60_Cl_2_Cu_3_), fw = 573.17 g∙mol^–1^, orthorhombic, space group *Pnnm*, *a* = 11.741(2) Å, *b* = 8.624(2) Å, *c* = 8.753(2) Å, *V* = 886.2(3) Å^3^, *Z* = 2, *μ* (Mo Kα, *λ* = 0.71073 Å) = 3.908 mm^–1^, T = 195(2) K, 7,875 reflections were collected, 1,077 were unique (*R*_int_ = 0.0603). One copper atom on a crystallographic 2/m symmetry site, the other copper atom and one bridging chloride on a crystallographic mirror plane and a tz ligand on general position are observed as an asymmetric unit. A CO_2_ site around a copper ion on the crystallographic mirror plane was identified in the difference Fourier map and its site occupancy factor was refined to 0.2001(7). All non-hydrogen atoms are refined anisotropically; the hydrogen atom attached to the carbon atom of the tz ligand was found in the difference Fourier map and refined isotropically. Least-squares refinement of the structure converged at a final *R*1 = 0.0397, *wR*2 = 0.0905 for 1043 reflections with *I* > 2*σ*(*I*); *R*1 = 0.0411, *wR*2 = 0.0911 for all 1077 reflections. The largest difference peak and hole were 0.892 and −0.950 e·Å^−3^, respectively.

(**CO_2_)_0.26_**(**H_2_O)_0.15_**@**1a-296K-5h.** [Cu_3_Cl_2_(tz)_4_(CO_2_)_0.26_]∙0.15H_2_O (C_4.26_H_4.30_N_16_O_0.67_Cl_2_Cu_3_), fw = 551.87 g∙mol^–1^, orthorhombic, space group *Pnnm*, *a* = 11.727(2) Å, *b* = 8.611(2) Å, *c* = 8.778(2) Å, *V* = 886.4(3) Å^3^, *Z* = 2, *μ* (Mo Kα, *λ* = 0.71073 Å) = 3.899 mm^–1^, T = 296(2) K, 7,928 reflections were collected, 1,077 were unique (*R*_int_ = 0.0445). One copper atom on a crystallographic 2/m symmetry site, the other copper atom and one bridging chloride on a crystallographic mirror plane and a tz ligand on general position are observed as an asymmetric unit. A CO_2_ site around a copper ion on the crystallographic mirror plane was identified in the difference Fourier map and was treated as statistically disordered CO_2_ and water sites. The site occupancy factors of the CO_2_ and the water sites were refined to 0.066(9) and 0.037(21), respectively. All non-hydrogen atoms are refined anisotropically; the hydrogen atom attached to the carbon atom of the tz ligand was found in the difference Fourier map and refined isotropically. Least-squares refinement of the structure converged at a final *R*1 = 0.0323, *wR*2 = 0.0754 for 1052 reflections with *I* > 2*σ*(*I*); *R*1 = 0.0329, *wR*2 = 0.0757 for all 1077 reflections. The largest difference peak and hole were 0.618 and −0.580 e·Å^−3^, respectively.

(**CO_2_)_0.12_(H_2_O)_0.50_@1a-296K-5d.** [Cu_3_Cl_2_(tz)_4_(CO_2_)_0.12_]∙0.50H_2_O (C_4.12_H_5.00_N_16_O_0.74_Cl_2_Cu_3_), fw = 552.02 g∙mol^–1^, orthorhombic, space group *Pnnm*, *a* = 11.722(2) Å, *b* = 8.612(2) Å, *c* = 8.780(2) Å, *V* = 886.3(3) Å^3^, *Z* = 2, *μ* (Mo Kα, *λ* = 0.71073 Å) = 3.899 mm^–1^, T = 296(2) K, 7,952 reflections were collected, 1,077 were unique (*R*_int_ = 0.0452). One copper atom on a crystallographic 2/m symmetry site, the other copper atom and one bridging chloride on a crystallographic mirror plane and a tz ligand on general position are observed as an asymmetric unit. A CO_2_ site around a copper ion on the crystallographic mirror plane was identified in the difference Fourier map and was treated as statistically disordered CO_2_ and water sites. The site occupancy factors of the CO_2_ and the water sites were refined to 0.030(8) and 0.124(23), respectively. All non-hydrogen atoms are refined anisotropically; the hydrogen atom attached to the carbon atom of the tz ligand was found in the difference Fourier map and refined isotropically. Least-squares refinement of the structure converged at a final *R*1 = 0.0323, *wR*2 = 0.0771 for 1060 reflections with *I* > 2*σ*(*I*); *R*1 = 0.0328, *wR*2 = 0.0774 for all 1077 reflections. The largest difference peak and hole were 0.647 and −0.649 e·Å^−3^, respectively.

(**H_2_O)_2_@1a-296K-10d.** [Cu_3_Cl_2_(tz)_4_(H_2_O)_0.76_]∙1.24H_2_O (C_4_H_8_N_16_O_2_Cl_2_Cu_3_), fw = 573.78 g∙mol^–1^, orthorhombic, space group *Pnnm*, *a* = 11.746(2) Å, *b* = 8.597(2) Å, *c* = 8.801(2) Å, *V* = 888.7(3) Å^3^, *Z* = 2, *μ* (Mo Kα, *λ* = 0.71073 Å) = 3.899 mm^–1^, T = 296(2) K, 8,043 reflections were collected, 1,081 were unique (*R*_int_ = 0.0418). One copper atom on a crystallographic 2/m symmetry site, the other copper atom and one bridging chloride on a crystallographic mirror plane and a tz ligand on general position are observed as an asymmetric unit. Two water sites around a copper ion on the crystallographic mirror plane were identified in the difference Fourier map and were treated as statistically disordered water sites. The site occupancy factors were refined to 0.189(4) and 0.311(4), respectively. All non-hydrogen atoms are refined anisotropically; the hydrogen atom attached to the carbon atom of the tz ligand was found in the difference Fourier map and refined isotropically. The hydrogen atoms attached to the water molecules were not included in the least-squares refinement. Refinement of the structure converged at a final *R*1 = 0.0264, *wR*2 = 0.0672 for 1046 reflections with *I* > 2*σ*(*I*); *R*1 = 0.0274, *wR*2 = 0.0677 for all 1081 reflections. The largest difference peak and hole were 0.555 and −0.421 e·Å^−3^, respectively.

A summary of the crystal data and some crystallography data is given in Tables S1-S6. CCDC-1492751-1492756 contain the supplementary crystallographic data for **1**, **1a**, (**CO_2_)_0.8_**@**1a-195K**, (**CO_2_)_0.12_(H_2_O)_0.50_@1a-296K-5d** and (**H_2_O)_2_@1a-296K-10d**. The data can be obtained free of charge at www.ccdc.cam.ac.uk/conts/retrieving.html or from the Cambridge Crystallographic Data Centre, 12 Union Road, Cambridge CB2 1EZ, UK.

Table S1. Crystal data and structure refinement for **1 ((MeOH)_2_@1a)**.

Empirical formula C_6_H_12_N_16_O_2_Cl_2_Cu_3_

Formula weight 601.84

Temperature 173(2) K

Wavelength 0.71073 Å

Crystal system Orthorhombic

Space group *Pnnm*

Unit cell dimensions a = 11.584(2) Å α = 90°

b = 8.5934(17) Å β = 90°

c = 8.8655(18) Å γ = 90°

Volume 882.5(3) Å^3^

Z 2

Density (calculated) 2.265 Mg/m^3^

Absorption coefficient 3.932 mm^-1^

F(000) 594

Crystal size 0.13 x 0.12 x 0.11 mm^3^

Theta range for data collection 3.30 to 27.45°.

Index ranges -12<=h<=15, -11<=k<=11, -11<=l<=11

Reflections collected 8176

Independent reflections 1075 [R(int) = 0.0275]

Completeness to theta = 27.45° 99.8 %

Absorption correction Semi-empirical from equivalents

Max. and min. transmission 0.6716 and 0.6289

Refinement method Full-matrix least-squares on F^2^

Data / restraints / parameters 1075 / 1 / 102

Goodness-of-fit on F^2^ 1.134

Final R indices [I>2sigma(I)] R1 = 0.0192, wR2 = 0.0449

R indices (all data) R1 = 0.0204, wR2 = 0.0455

Largest diff. peak and hole 0.416 and −0.524 e·Å^−3^

Table S2. Crystal data and structure refinement for **1a**.

Empirical formula C_4_ H_4_N_16_Cl_2_Cu_3_

Formula weight 537.75

Temperature 393(2) K

Wavelength 0.62999 Å

Crystal system Orthorhombic

Space group *Pnnm*

Unit cell dimensions a = 11.729(2) Å α = 90°

b = 8.5910(17) Å β = 90°

c = 8.7650(18) Å γ = 90°

Volume 883.2(3) Å^3^

Z 2

Density (calculated) 2.022 Mg/m^3^

Absorption coefficient 2.814 mm^-1^

F(000) 522

Crystal size 0.12 x 0.12 x 0.10 mm^3^

Theta range for data collection 3.73 to 29.49°.

Index ranges −18<=h<=18, −12<=k<=12, −13<=l<=13

Reflections collected 11893

Independent reflections 1774 [R(int) = 0.0454]

Completeness to theta = 29.49° 94.7 %

Absorption correction Semi-empirical from equivalents

Max. and min. transmission 0.7661 and 0.7288

Refinement method Full-matrix least-squares on F^2^

Data / restraints / parameters 1774 / 0 / 68

Goodness-of-fit on F^2^ 1.174

Final R indices [I>2sigma(I)] R1 = 0.0411, wR2 = 0.1117

R indices (all data) R1 = 0.0445, wR2 = 0.1214

Extinction coefficient 0.24(2)

Largest diff. peak and hole 1.640 and −1.687 e·Å^−3^

Table S3. Crystal data and structure refinement for (**CO_2_)_0.8_@1a-195K**.

Empirical formula C_4.8_H_4_N_16_O_1.60_Cl_2_Cu_3_

Formula weight 572.93

Temperature 195(2) K

Wavelength 0.71073 Å

Crystal system Orthorhombic

Space group *Pnnm*

Unit cell dimensions a = 11.741(2) Å α = 90°

b = 8.6235(17) Å β = 90°

c = 8.7530(18) Å γ = 90°

Volume 886.2(3) Å^3^

Z 2

Density (calculated) 2.150 Mg/m^3^

Absorption coefficient 3.908 mm^-1^

F(000) 558

Crystal size 0.35 x 0.27 x 0.24 mm^3^

Theta range for data collection 3.32 to 27.46°.

Index ranges −14<=h<=15, −11<=k<=11, −11<=l<=11

Reflections collected 7875

Independent reflections 1077 [R(int) = 0.0603]

Completeness to theta = 27.46° 99.7 %

Absorption correction Semi-empirical from equivalents

Max. and min. transmission 0.4539 and 0.3416

Refinement method Full-matrix least-squares on F^2^

Data / restraints / parameters 1077 / 0 / 85

Goodness-of-fit on F^2^ 1.149

Final R indices [I>2sigma(I)] R1 = 0.0397, wR2 = 0.0905

R indices (all data) R1 = 0.0411, wR2 = 0.0911

Largest diff. peak and hole 0.892 and −0.950 e·Å^−3^

Table S4. Crystal data and structure refinement for (**CO_2_)_0.26_**(**H_2_O)_0.15_@1a-296K-5h**.

Empirical formula C_4.26_H_4.3_N_16_O_0.67_Cl_2_Cu_3_

Formula weight 551.87

Temperature 296(2) K

Wavelength 0.71073 Å

Crystal system Orthorhombic

Space group *Pnnm*

Unit cell dimensions a = 11.727(2) Å α = 90°

b = 8.6111(17) Å β = 90°

c = 8.7780(18) Å γ = 90°

Volume 886.4(3) Å^3^

Z 2

Density (calculated) 2.067 Mg/m^3^

Absorption coefficient 3.899 mm^-1^

F(000) 536

Crystal size 0.35 x 0.27 x 0.24 mm^3^

Theta range for data collection 3.31 to 27.44°.

Index ranges −14<=h<=15, −11<=k<=11, −11<=l<=11

Reflections collected 7928

Independent reflections 1077 [R(int) = 0.0445]

Completeness to theta = 27.44° 99.8 %

Absorption correction Semi-empirical from equivalents

Max. and min. transmission 0.4547 and 0.3423

Refinement method Full-matrix least-squares on F^2^

Data / restraints / parameters 1077 / 36 / 90

Goodness-of-fit on F^2^ 1.232

Final R indices [I>2sigma(I)] R1 = 0.0323, wR2 = 0.0754

R indices (all data) R1 = 0.0329, wR2 = 0.0757

Largest diff. peak and hole 0.618 and −0.580 e·Å^−3^

Table S5. Crystal data and structure refinement for (**CO_2_)_0.12_(H_2_O)_0.50_@1a-296K-5d**.

Empirical formula C_4.12_H_5_N_16_O_0.72_Cl_2_Cu_3_

Formula weight 551.74

Temperature 296(2) K

Wavelength 0.71073 Å

Crystal system Orthorhombic

Space group *Pnnm*

Unit cell dimensions a = 11.722(2) Å α = 90°

b = 8.6124(17) Å β = 90°

c = 8.7797(18) Å γ = 90°

Volume 886.3(3) Å^3^

Z 2

Density (calculated) 2.071 Mg/m^3^

Absorption coefficient 3.899 mm^-1^

F(000) 538

Crystal size 0.35 x 0.27 x 0.24 mm^3^

Theta range for data collection 3.31 to 27.44°.

Index ranges −14<=h<=15, −11<=k<=11, −11<=l<=11

Reflections collected 7952

Independent reflections 1077 [R(int) = 0.0452]

Completeness to theta = 27.44° 99.8 %

Absorption correction Semi-empirical from equivalents

Max. and min. transmission 0.4546 and 0.3423

Refinement method Full-matrix least-squares on F^2^

Data / restraints / parameters 1077 / 30 / 90

Goodness-of-fit on F^2^ 1.220

Final R indices [I>2sigma(I)] R1 = 0.0323, wR2 = 0.0771

R indices (all data) R1 = 0.0328, wR2 = 0.0774

Largest diff. peak and hole 0.647 and −0.649 e·Å^−3^

Table S6. Crystal data and structure refinement for **(H_2_O)_2_@1a-296K-10d**.

Empirical formula C_4_H_8_N_16_O_2_Cl_2_Cu_3_

Formula weight 573.78

Temperature 296(2) K

Wavelength 0.71073 Å

Crystal system Orthorhombic

Space group *Pnnm*

Unit cell dimensions a = 11.746(2) Å α = 90°

b = 8.5973(17) Å β = 90°

c = 8.8011(18) Å γ = 90°

Volume 888.7(3) Å^3^

Z 2

Density (calculated) 2.144 Mg/m^3^

Absorption coefficient 3.899 mm^-1^

F(000) 562

Crystal size 0.35 x 0.27 x 0.24 mm^3^

Theta range for data collection 3.31 to 27.45°.

Index ranges -15<=h<=14, -11<=k<=11, -11<=l<=11

Reflections collected 8043

Independent reflections 1081 [R(int) = 0.0418]

Completeness to theta = 27.45° 99.8 %

Absorption correction Semi-empirical from equivalents

Max. and min. transmission 0.4547 and 0.3423

Refinement method Full-matrix least-squares on F^2^

Data / restraints / parameters 1081 / 1 / 80

Goodness-of-fit on F^2^ 1.124

Final R indices [I>2sigma(I)] R1 = 0.0264, wR2 = 0.0672

R indices (all data) R1 = 0.0274, wR2 = 0.0677

Largest diff. peak and hole 0.555 and −0.421 e·Å^−3^


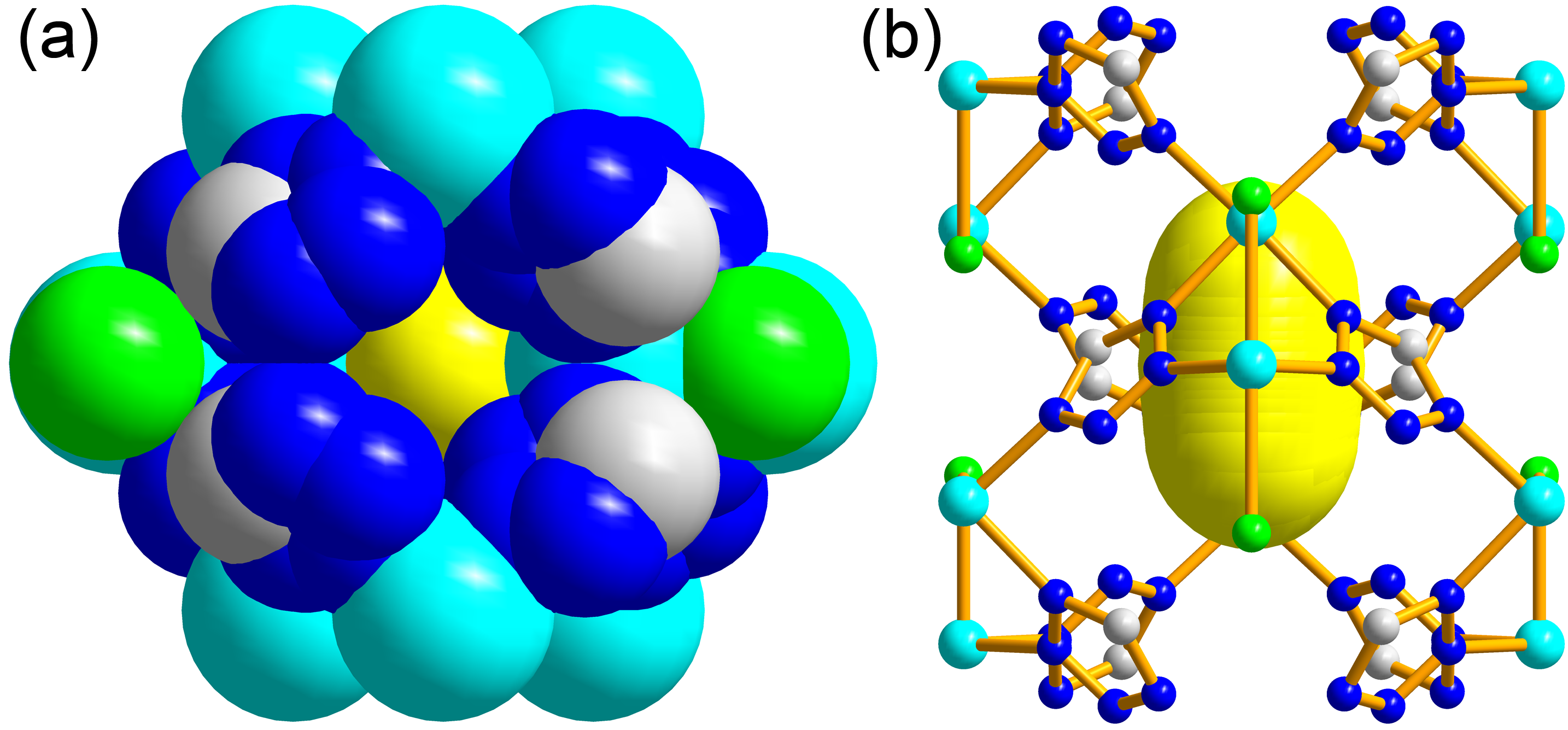


Figure S1. (a) The space-filling diagram of the ellipsoidal cage showing one of the two portals viewed along the crystallographic *b*-axis. (b) The ball-and-stick diagram of the pore with an ellipsoidal cavity viewed along the crystallographic *c*-axis. The yellow dummy ellipsoid represents the cavity space in the center of the cage.

**Crystal structure of (MeOH)_2_@1a.** The oxygen atom of the ligated methanol molecule is hydrogen-bonded to the oxygen atom of the symmetry related ligated methanol in the cage (Table S7). The oxygen atom of the statistically disordered lattice methanol molecule in the vicinity of the ligated methanol molecules is also in close contact (or in hydrogen-bonding interaction) to the oxygen atom of the symmetry related lattice methanol molecule (Figure S2).

Table S7. Hydrogen bonds for **1** (Å and °).

___________________________________________________________________________

D-H...A d(D-H) d(H...A) d(D...A) <(DHA)

___________________________________________________________________________

O(1M)-H(1M)...O(1M)#1 0.84 2.20 2.988(9) 155.5

O(2M)...O(2M)#1 2.955(11)

___________________________________________________________________________

Symmetry transformations used to generate equivalent atoms: #1 -x+1,-y+2,-z+2


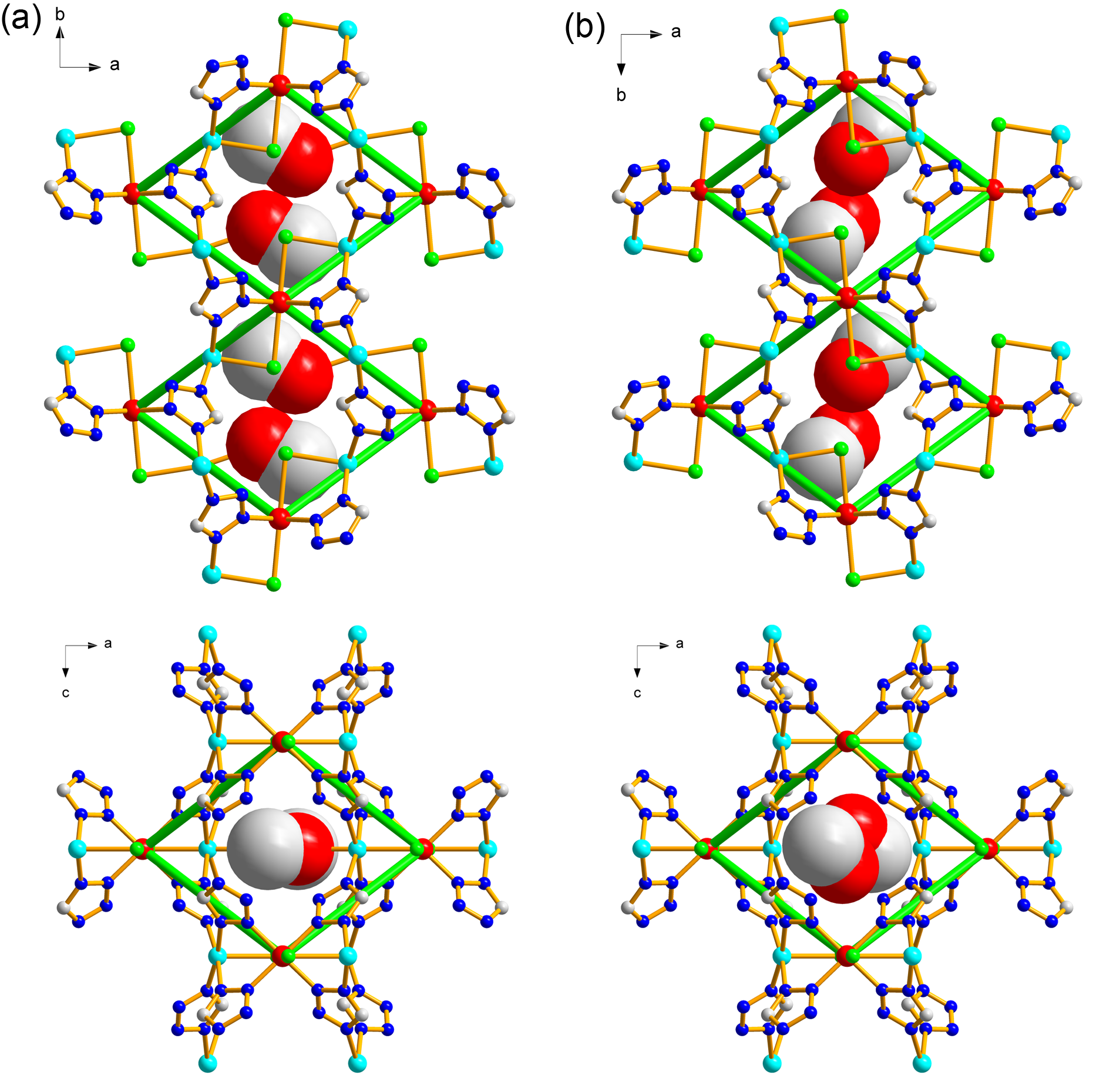


Figure S2. A 1-D porous channel made of cages viewed down along the crystallographic *c*-axis and the crystallographic *b*-axis, respectively. (a) The ligated methanol molecules in the cages are hydrogen-bonded to each other. (b) The statistically disordered two lattice methanol molecules in the vicinity of the ligated methanol molecules are in close contact to each other or in hydrogen bonding interaction with the ligated methanol molecules in the same cage.

**TGA of (MeOH)_2_@1a.** The TGA of (**MeOH)_2_@1a** shows multi-step weight losses up to 600 °C (Figure S3). Although the EA and the single crystal structure analysis of (**MeOH)_2_@1a** indicated the presence of two equivalent amounts of removable methanol in the cage-like solvent pore of the 1-D solvent channel, the weight loss up to ~ 240 °C (4.2%) was much smaller than the calculated methanol amount in the solvent cavity (10.6%). The significant weight loss up to 270 °C (~ 25%) indicated that the complete removal of the methanol molecules in the cage only occurs together with the decomposition of the ligand and the framework.


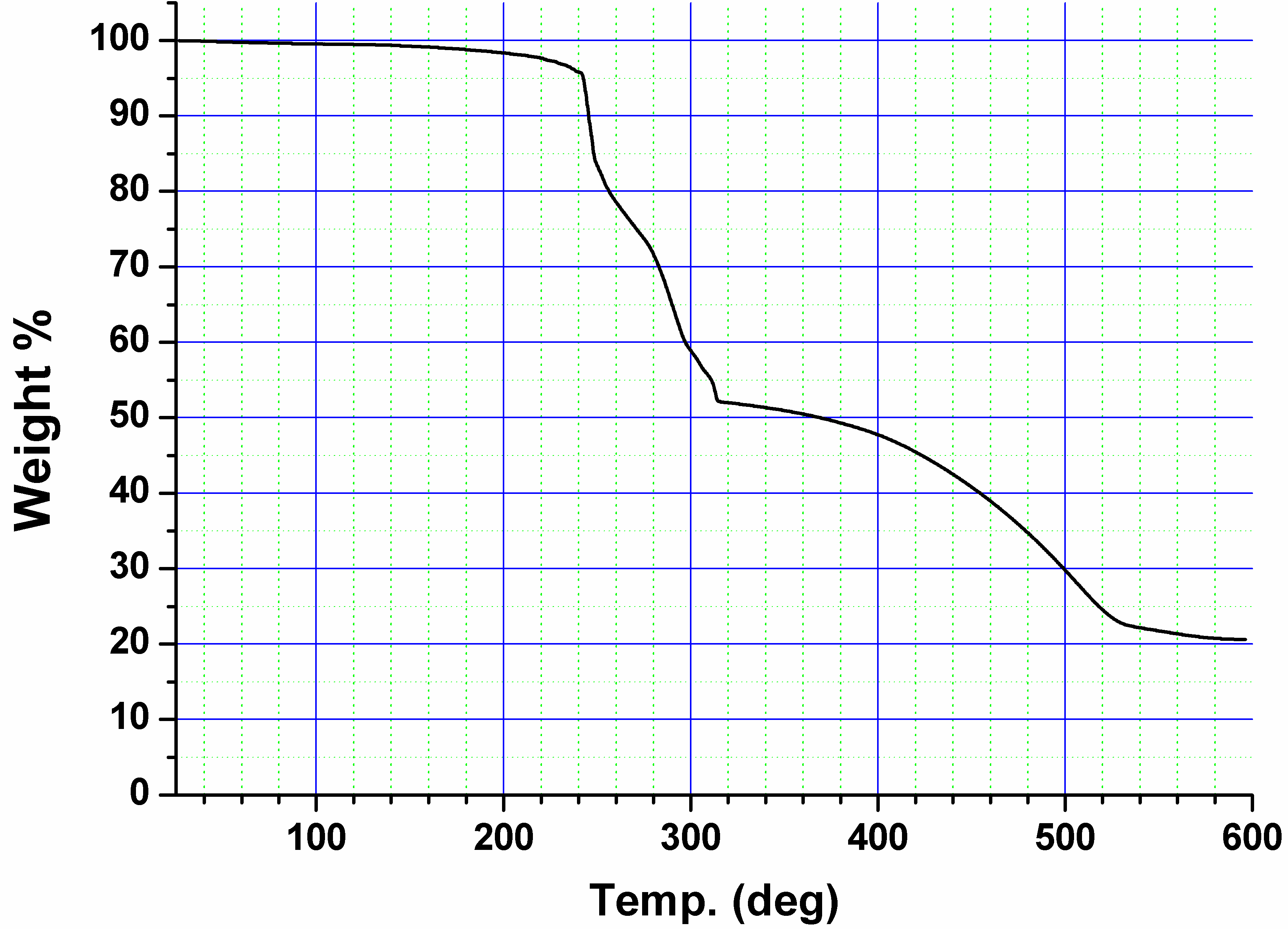


Figure S3. TGA of (**MeOH)_2_@1a**.

**PXRD of (MeOH)_2_@1a.** Although (**MeOH)_2_@1a** is thermally stable at 200 °C for 1 h under N_2_, the PXRD pattern of (**MeOH)_2_@1a** activated at 200 °C for 1 d under vacuum (~ 10^−2^ torr) showed several additional peaks corresponding to the decay product in addition to the diffraction peaks of (**MeOH)_2_@1a** (Figure S4).


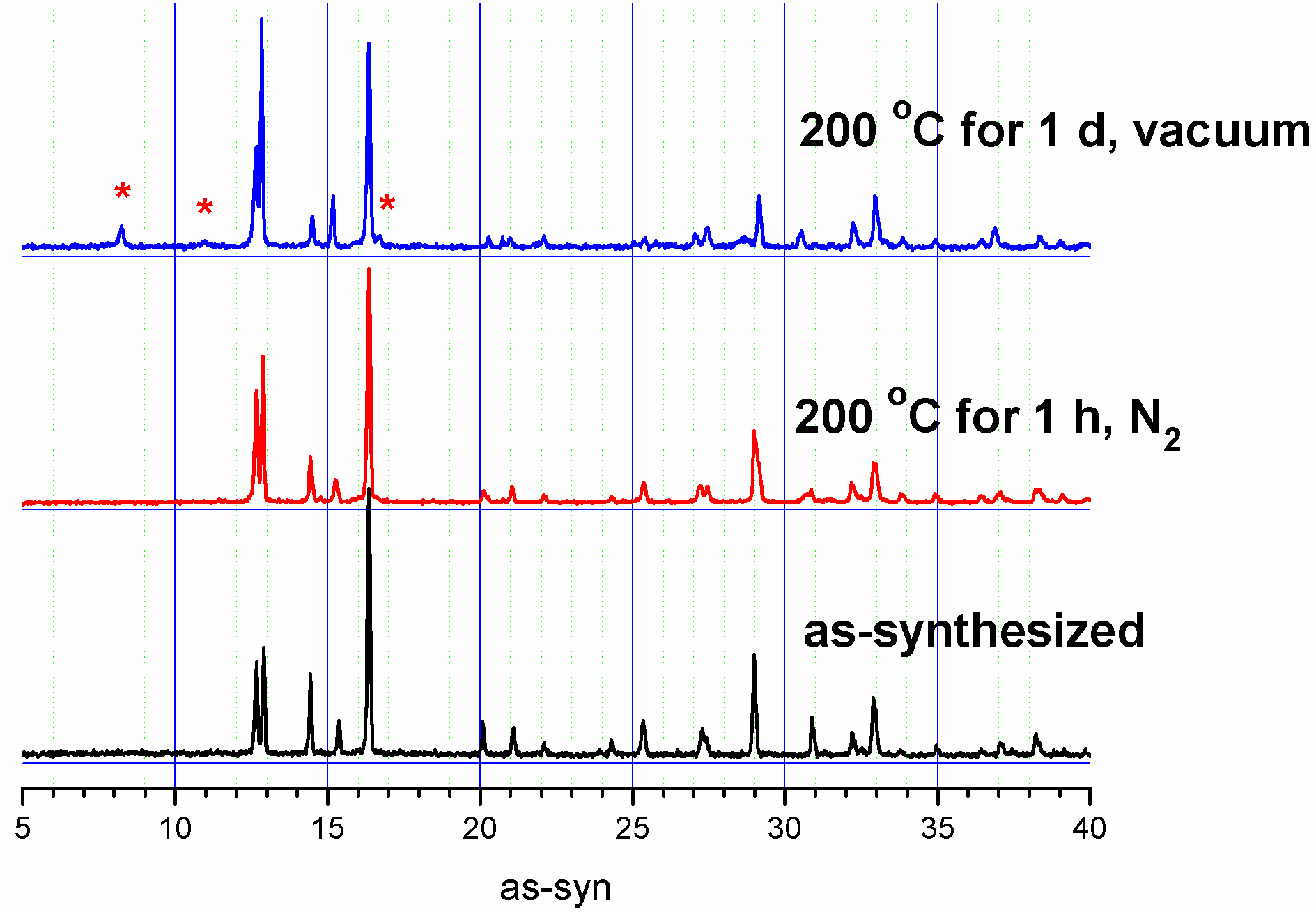


Figure S4. The PXRD patterns of (**MeOH)_2_@1a** thermally treated at 200 °C for 1 h under N_2_, and activated at 200 °C for 1 d under vacuum, respectively.

The properly activated sample, **1a**, was prepared by vacuuming (~ 10^−2^ torr) (**MeOH)_2_@1a** at 160 °C for 7 d. The PXRD pattern of **1a** neither indicated any loss of the crystallinity nor the appearance of new additional diffraction peaks corresponding to the decay product (Figure S5).


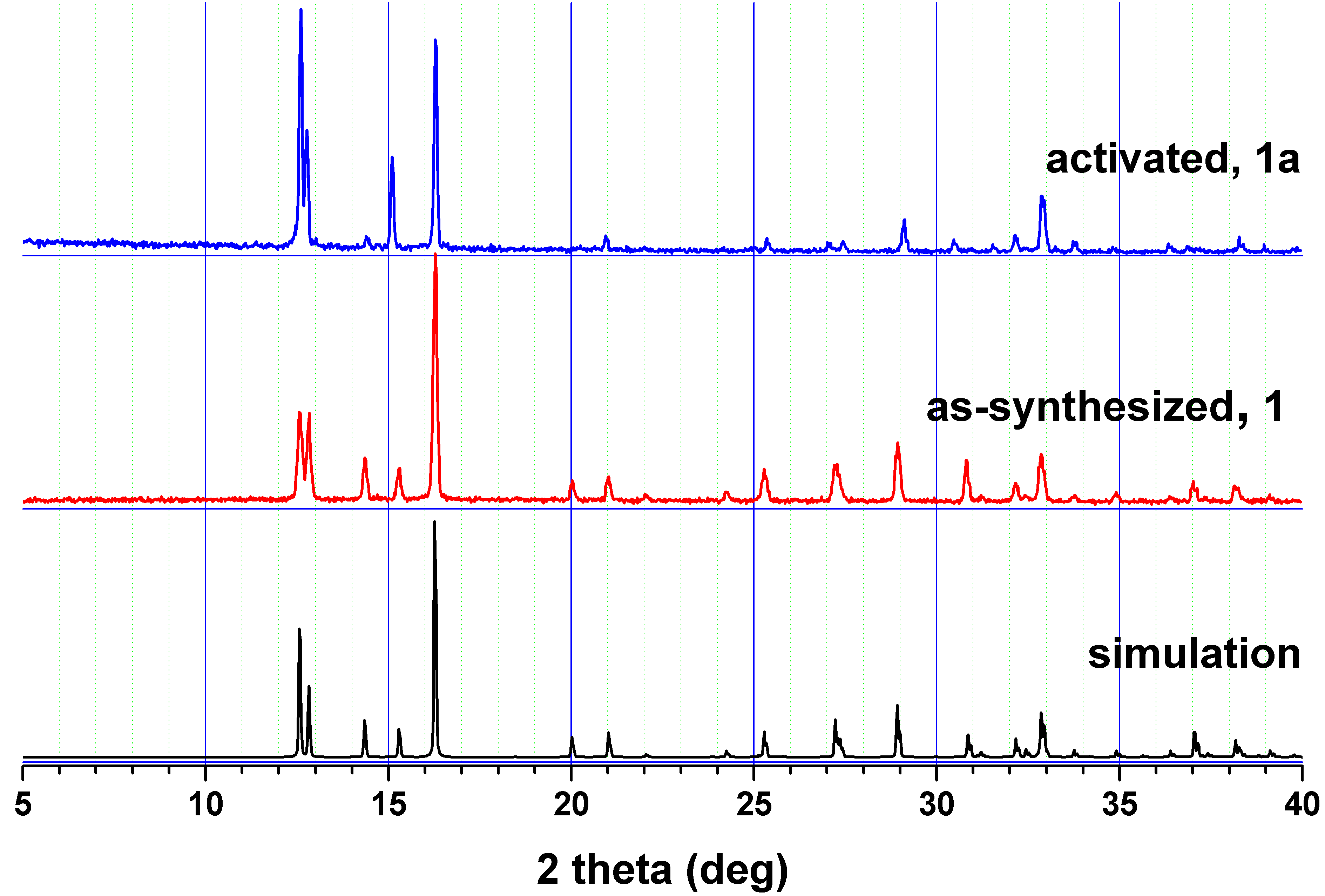


Figure S5. The PXRD pattern of **1a**.

**^1^H NMR spectra** **of (MeOH)_2_@1a and 1a.** The ^1^H NMR spectrum of **1a** digested in DCl/DMSO-*d*_6_ solvent confirmed the removal of the most solvent methanol molecules from the solvent pore. While the ^1^H NMR spectrum of (**MeOH)_2_@1a** digested in DCl/DMSO-*d*_6_ solvent showed ~ 2 equivalent amounts of methanol per formula unit of the framework, [Cu_3_Cl_2_(tz)_4_] (C*H* of tz:–OC*H*_3_ of MeOH = 1:1.50), only a very small amount of solvent methanol peaks ((C*H* of tz:–OC*H*_3_ of MeOH = 1:0.08)) are remaining in the corresponding ^1^H NMR spectrum of **1a** (Figure S6).


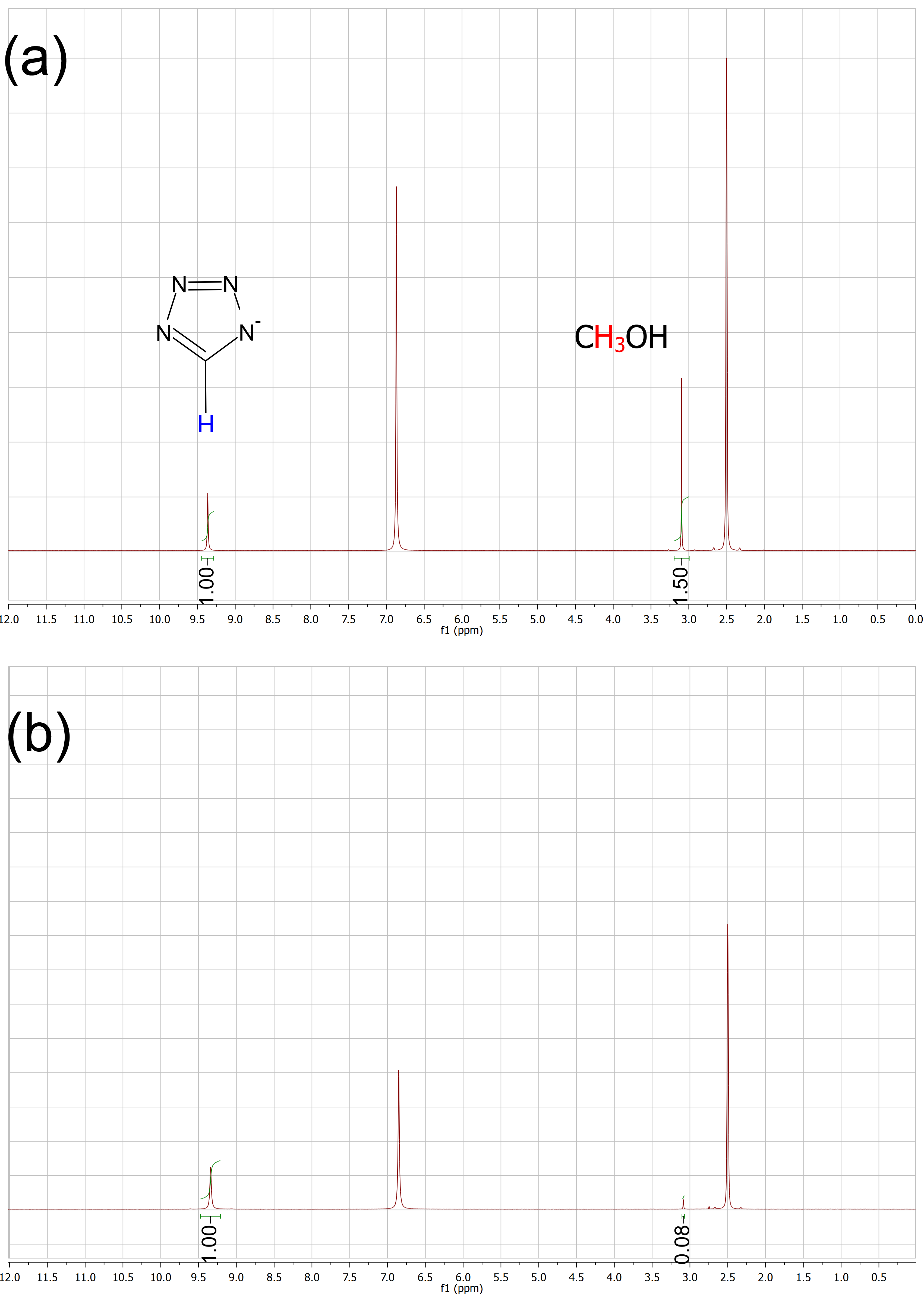


Figure S6. The ^1^H NMR spectra of (a) (**MeOH)_2_@1a** and (b) **1a** digested in in DCl/ *d*_6_-DMSO.

**Crystal structure of 1a.**


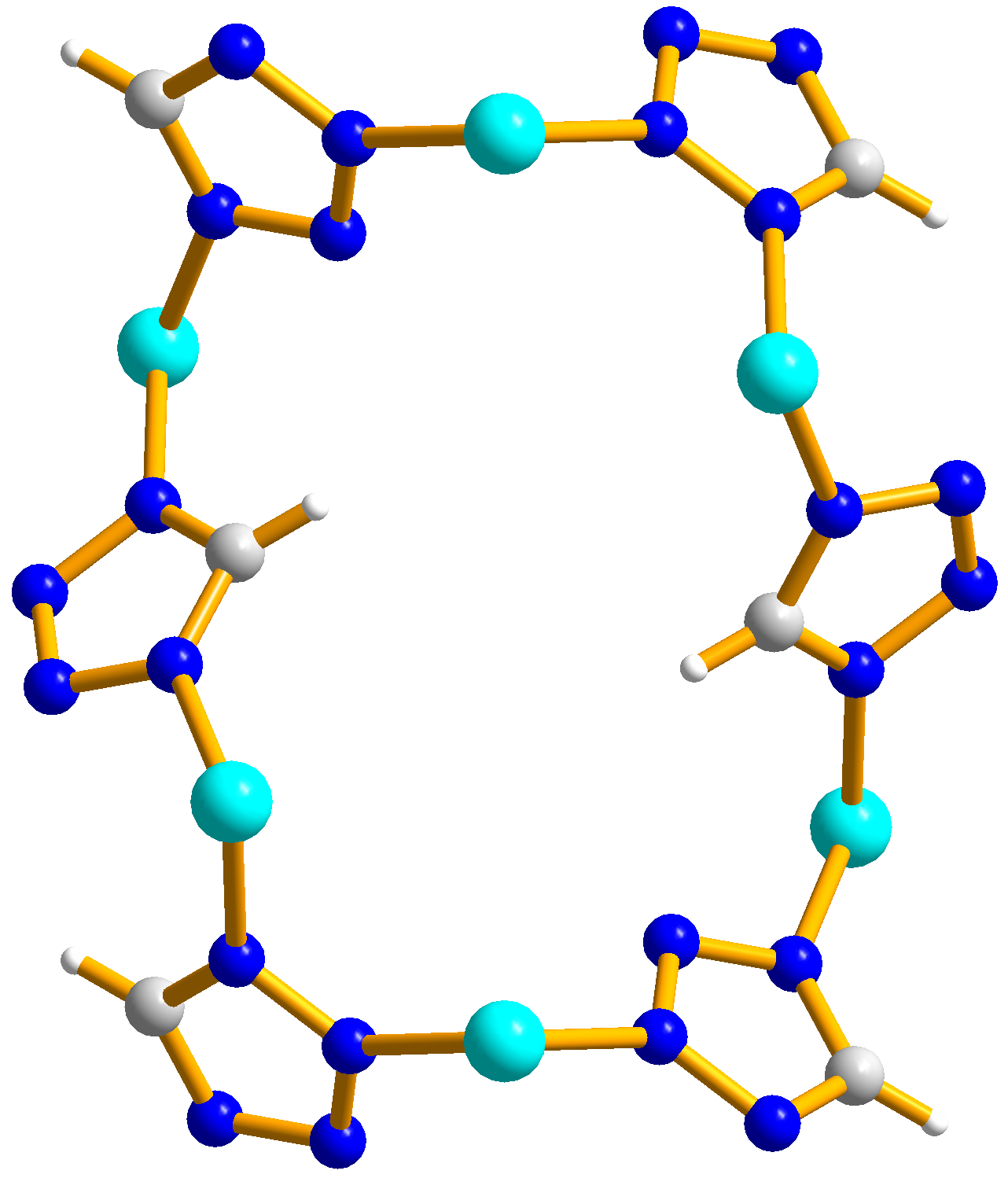


Figure S7. The single crystal structure of **1a**.

**Crystal structures of** (**CO_2_)_0.12_(H_2_O)_0.50_@1a-296K-5d and** (**H_2_O)_2_@1a-296K-10d.** After letting the single crystal of (**CO_2_)_0.8_@1a-195K** at ambient condition for five days, the crystal of (**CO_2_)_0.12_(H_2_O)_0.50_@1a-296K-5d** (the crystal of **CO_2_@1a-195K** stood at ambient condition for five days) still contains some bound CO_2_ molecules in the cage but its site occupancy is further reduced to 0.06(2) (Figure S8 and Table S8). The complete replacement of the bound CO_2_ molecules in the pore by the water molecules in air took 10 days at ambient condition. A single crystal structure of (**H_2_O)_2_@1a-296K-10d** (the single crystal of **CO_2_@1a-195K** stood at ambient condition for 10 days) showed only the weakly bound H_2_O molecules at the cage with site occupancy, 0.378(8) (Figure S9).


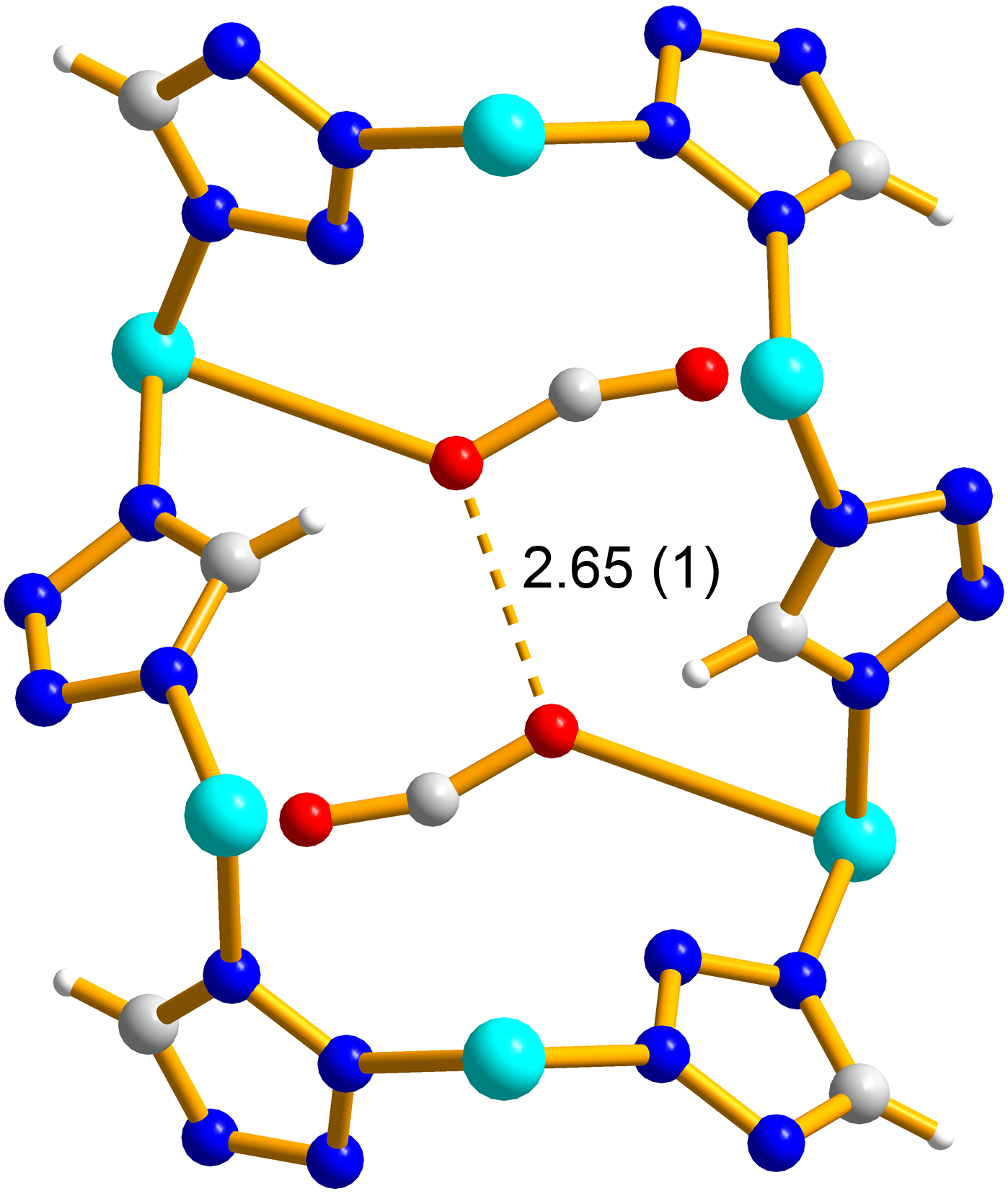


Figure S8. The single crystal structure of (**CO_2_)_0.12_(H_2_O)_0.50_@1a-296K-5d**. The statistically disordered two lattice water molecule sites in the vicinity of the ligated CO_2_ molecules are omitted for clarity.


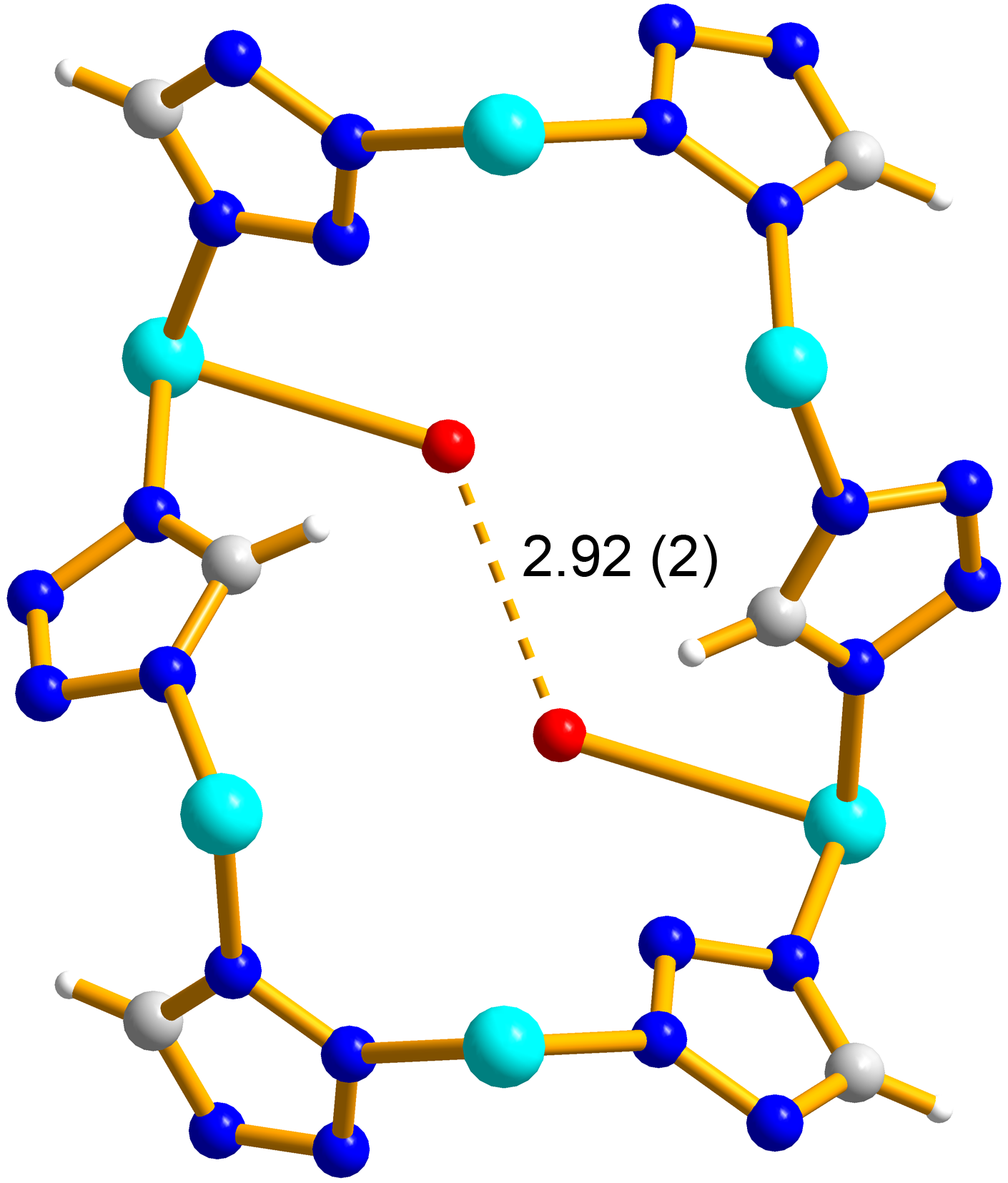


Figure S9. The single crystal structures of (**H_2_O)_2_@1a-296K-10d**. The statistically disordered two lattice water molecule sites in the vicinity of the ligated water molecules are omitted for clarity.

Table S8. Selected distances and angles (Å and °) around the bound CO_2_ or guest molecules in MOFs.

|  | # of bound molecules per M site | Cu2−Cl1 | Cu2−O1C | Cl1−Cu2−O1C | O1C−C1C | C1C−O2C | O1C−C1C−O2C | Cu2−O1C−C1C | N3 … C1C | Ref. |
| --- | --- | --- | --- | --- | --- | --- | --- | --- | --- | --- |
| **(MeOH)_2_@1a** | 0.50(1) | 2.487(1) | 2.617(5) | 170.4(1) | 1.43(2) | - | - | 131.4(6) | - | This work |
| **1a** | - | 2.412(1) | - | - | - | - | - | - | - | This work |
| **(CO_2_)_0.8_@1a-195K** | 0.40(1) | 2.426(2) | 2.94(2) | 167.6(3) | 1.12(3) | 1.13(3) | 176(3) | 124(2) | 3.21(2) | This work |
| **(CO_2_)_0.52_(H_2_O)_0.3_@1a-296K-5h** | 0.13(2) | 2.419(1) | 3.09(5) | 169.9(9) | 1.16(7) | 1.03(9) | 172(7) | 124(4) | 3.34(4) | This work |
| **(CO_2_)_0.52_(H_2_O)_0.22_@1a-296K-1d** | 0.13(2) | 2.418(1) | 3.11(6) | 167(1) | 1.13(8) | 0.96(9) | 172(10) | 125(5) | 3.34(5) | This work |
| **(CO_2_)_0.22_(H_2_O)_1.0_@1a-296K-5d** | 0.06(2) | 2.418(1) | 3.01(7) | 168(1) | 1.28(8) | 1.19(10) | 160(6) | 131(4) | 3.38(3) | This work |
| **(H_2_O)_2_@1a-296K-10d** | 0.38(1) | 2.447(1) | 2.81(1) | 172.1(3) | - | - | - | - | - | This work |
| **(CO_2_)_0.8_@1a** | 0.67(1) |  | 2.29(2) | 168.1(7) | 1.21^*^ | 1.21^*^ | 162(3) | 117(2) | - | S6 |
| **Mg-MOF-74** | 0.24(1) |  | 2.39(6) |  | 1.1(1) | 1.04(9) | 150(15) | 125(7) | - | S7 |
| **Mg-MOF-74** | 0.60(1) |  | 2.34(3) |  | 1.07(5) | 1.06(4) | 164(6) | 128(3) | - | S7 |
| **Mg-MOF-74** | 0.89(1) |  | 2.30(2) |  | 1.17(3) | 1.06(2) | 172(4) | 129(2) | - | S7 |
| **Mg-MOF-74** | 0.88(1) |  | 2.28(3) |  | 1.13(4) | 1.10(3) | 167(4) | 134(2) | - | S7 |
| **Mg-MOF-74** | 0.99(1) |  | 2.24(3) |  | 1.14(3) | 1.06(3) | 170(3) | 144(2) | - | S7 |
| **Free CO_2_** |  |  |  |  | 1.155(1) |  | 180 |  | - | S8 |

^*^ The C–O distances within the CO2 molecule were constrained.

**Vibrational spectra of the CO_2_ molecule encapsulated in a cage.**


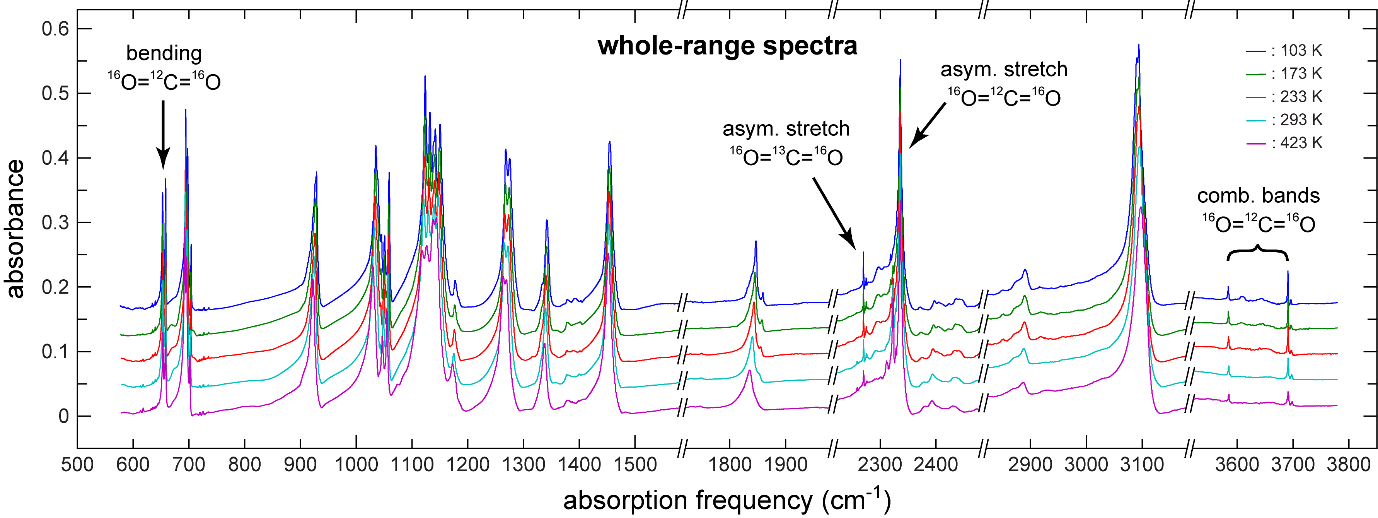


Figure S10. Whole-range spectra of **1a** filled with ^12^CO_2_ at 5 representative temperatures (103, 173, 233, 293 and 423 K). Peaks originating from the bending, asymmetric stretch, and combination bands of ^12^CO_2_ are marked with indication. The peak at ~ 2275 cm^-1^ originates from the asymmetric stretch of the ^13^CO_2_ coming from the natural abundance of ^13^C.


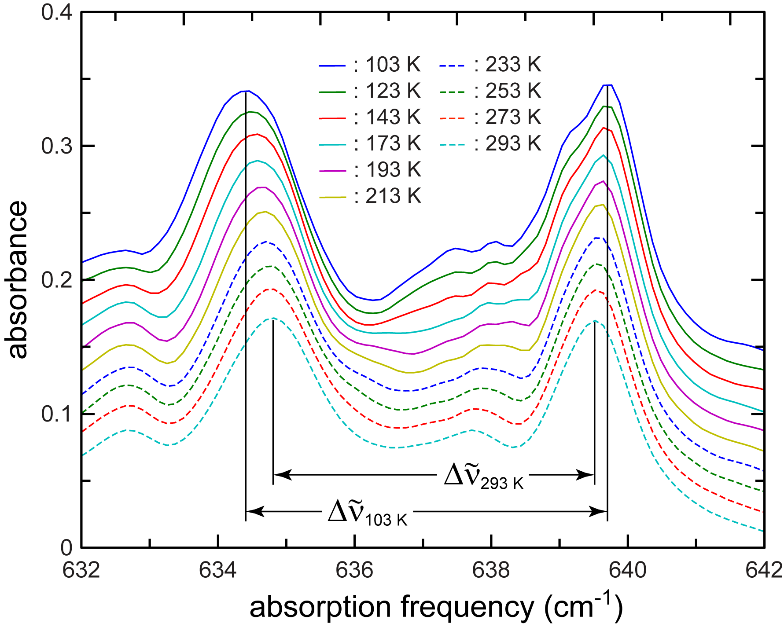


Figure S11. An enlarged view of the bending region of FTIR spectra of **1a** filled with ^13^CO_2_ at various temperatures. Δ$\tilde{\nu}$_103 K_ (5.30 cm^-1^) and Δ$\tilde{\nu}$_293 K_ (4.71 cm^-1^) represent the separation between the two peaks at 103 and 293 K, respectively.


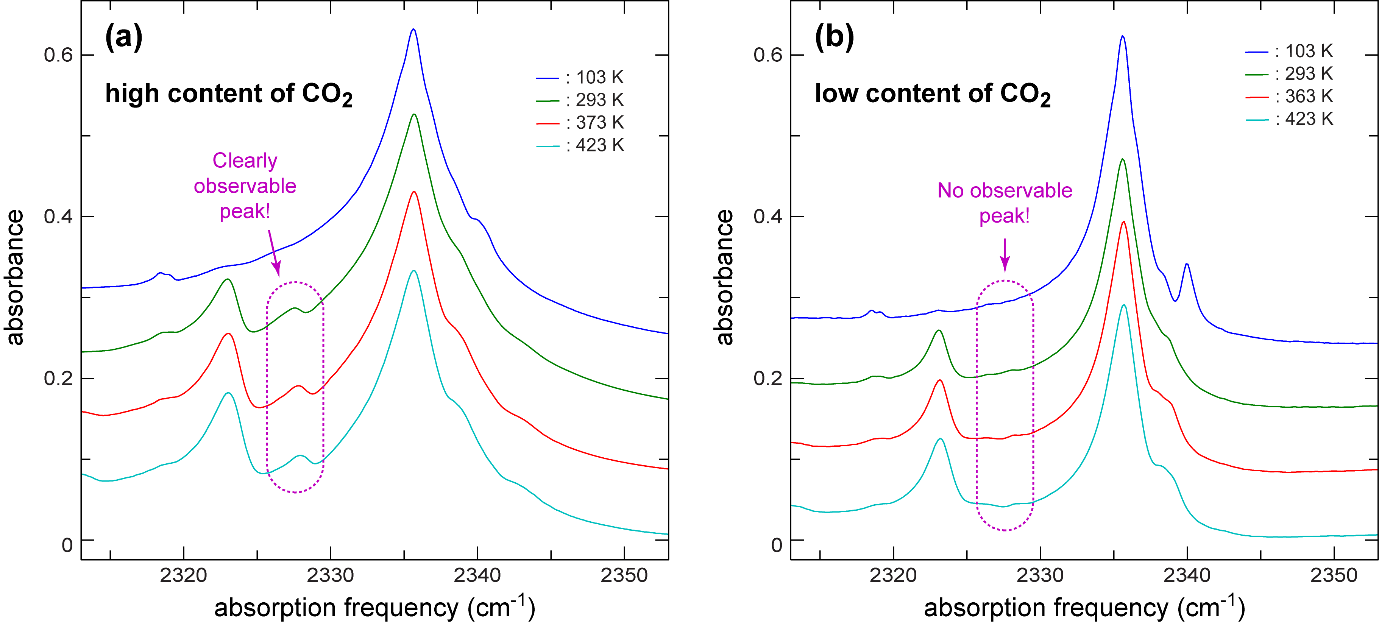


Figure S12. Temperature-dependent FTIR spectra of **1a** containing different amount of ^12^CO_2_ molecules in the spectral region of the CO_2_ asymmetric stretch at 103 (blue), 293 (green), 373 (red), and 423 (cyan) K, respectively. The rounded rectangles represent the spectral region where the CO_2_ double-occupancy peak is expected to appear. (a) FTIR spectra of **1a** with high content of CO_2_. The average CO_2_ occupancy per cage is estimated to be ~ 0.9 from the CO_2_ sorption study. (b) FTIR spectra of **1a** with low content of CO_2_. The average CO_2_ occupancy per cage is estimated to be ~ 0.3 from the comparison between the integrated area of the asymmetric stretch band in (a) and that in (b).

Gas sorption measurements. All gas sorption isotherms were measured using a BELSORP-max (BEL Japan, Inc.) with a standard volumetric technique using N_2_ (with purity of 99.999%), H_2_ (99.9999%), CH_4_ (99.95%), and CO_2_ (99.999%) as adsorbates. Even though 1a had microporous 1-D channel, it does not show any N_2_ (at 77 K and 308 K) and CH_4_ (at 195 K and 308 K) adsorptions (Figure S13). It is due to the large kinetic diameters of N_2_ and CH_4_ (3.64 Å and 3.8 Å, respectively) compared to the small portal dimension, 2.5 Å, between the cages interlinked to form microporous 1-D channels. 1a does not adsorb even H_2_ of the kinetic diameter, 2.89 Å, at 77 K.


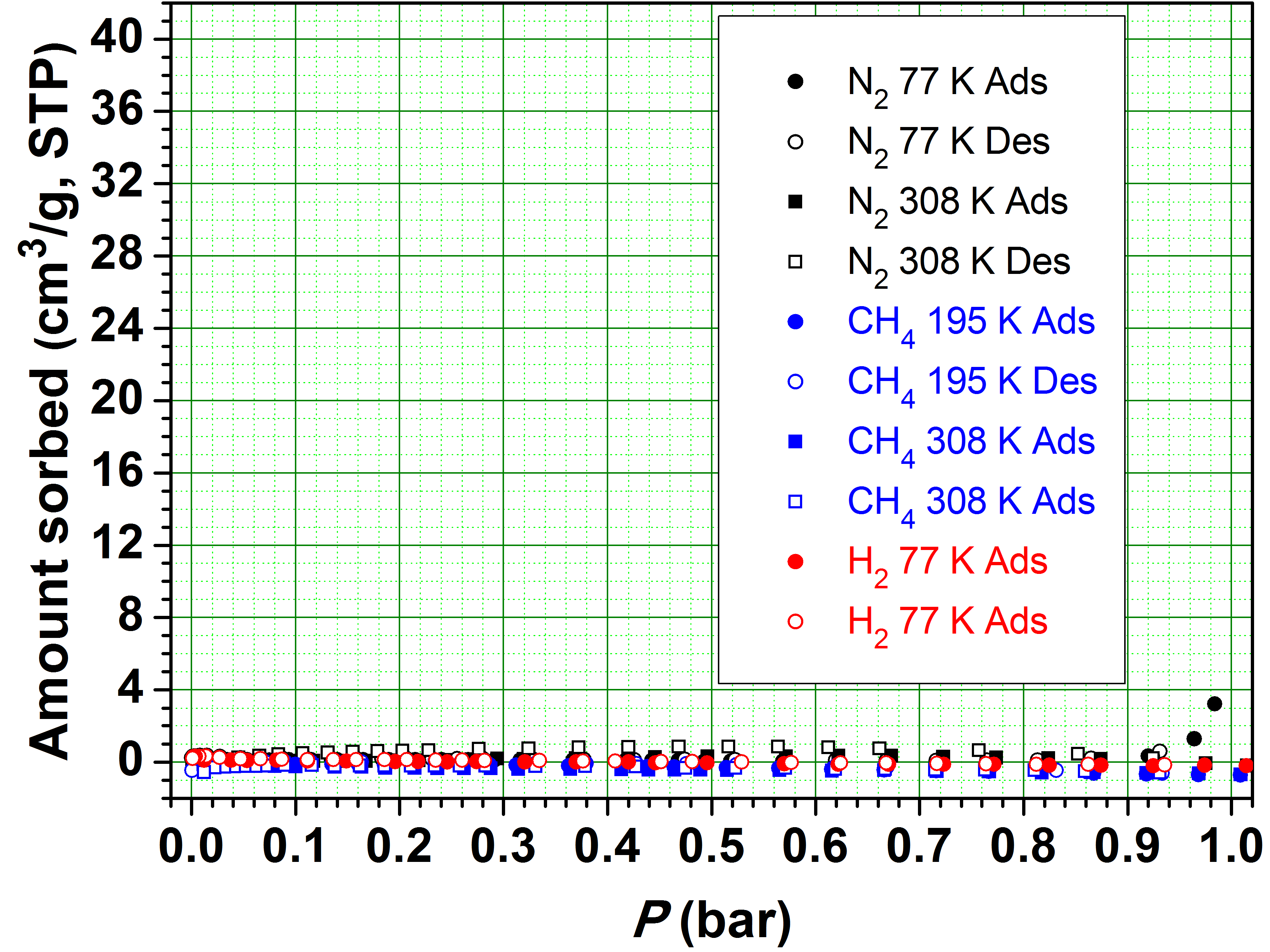


Figure S13. Gas sorption of 1a. N_2_ adsorption and desorption sorption isotherms at 77 K and 308 K, respectively; CH_4_ adsorption and desorption isotherms at 195 K and 308 K, respectively; H_2_ adsorption and desorption isotherms at 77 K.


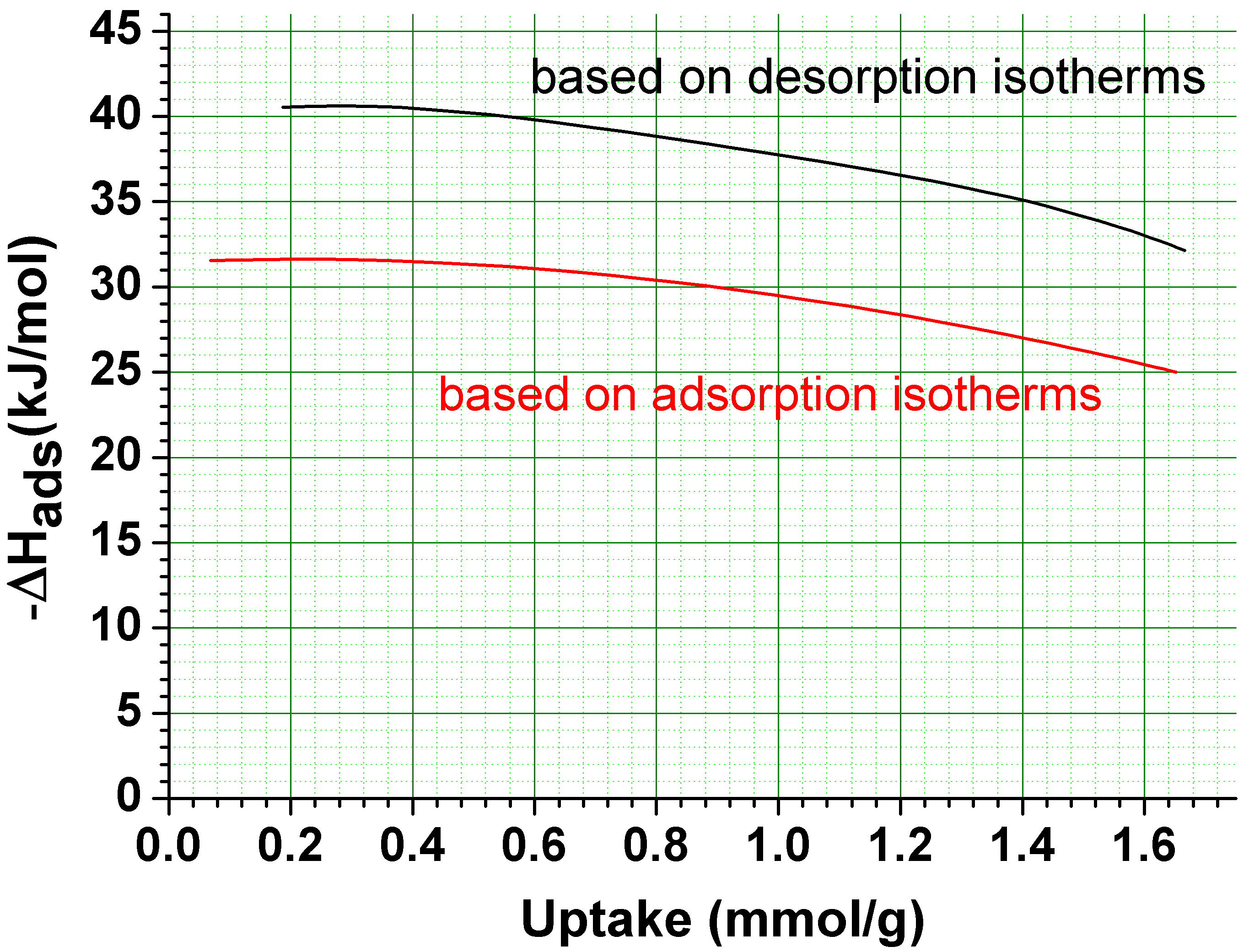


Figure S14. Adsorption enthalpy of CO_2_ on **1a**.

References

S1. Materials Studio program, version 4.3, Accelrys, San Diego, CA (2008).

S2. Rapid Auto software, R-Axis series, Cat. No. 9220B101, Rigaku Corporation.

S3. Arvai, A. J. & Nielsen, C. ADSC Quantum-210 ADX Program, Area Detector System Corporation; Poway, CA, USA (1983).

S4. SHELX program: Sheldrick, G. M. Crystal structure refinement with SHELXL. *Acta Crystallogr. Sect. C* **71**, 3 (2015).

S5. PLATON program: Spek, A. L. PLATON SQUEEZE: a tool for the calculation of the disordered solvent contribution to the calculated structure factors. *Acta Crystallogr. Sect. C* **71**, 9 (2015).

S6. Dietzel, P. D. C. *et al*. Adsorption properties and structure of CO_2_ adsorbed on open coordination sites of metal–organic framework Ni_2_(dhtp) from gas adsorption, IR spectroscopy and X-ray diffraction. *Chem. Commun.* 5125 (2008).

S7. Queen, W. L. *et al*. Site-specific CO_2_ adsorption and zero thermal expansion in an anisotropic pore network. *J. Phys. Chem. C* 115, 24915 (2011).

S8. Simon, A. & Peters, K. Single-crystal refinement of the structure of carbon dioxide. *Acta Cryst.* *B* 36, 2750 (1980).
